# Recurrent Loss of Specific Introns during Angiosperm Evolution

**DOI:** 10.1371/journal.pgen.1004843

**Published:** 2014-12-04

**Authors:** Hao Wang, Katrien M. Devos, Jeffrey L. Bennetzen

**Affiliations:** 1 Department of Genetics, University of Georgia, Athens, Georgia, United States of America; 2 Department of Crop and Soil Sciences, and Department of Plant Biology, University of Georgia, Athens, Georgia, United States of America; 3 Germplasm Bank of Wild Species in Southwestern China, Kunming Institute of Botany, Kunming, Yunnan, P.R. China; University of Utah School of Medicine, United Stated of America

## Abstract

Numerous instances of presence/absence variations for introns have been documented in eukaryotes, and some cases of recurrent loss of the same intron have been suggested. However, there has been no comprehensive or phylogenetically deep analysis of recurrent intron loss. Of 883 cases of intron presence/absence variation that we detected in five sequenced grass genomes, 93 were confirmed as recurrent losses and the rest could be explained by single losses (652) or single gains (118). No case of recurrent intron gain was observed. Deep phylogenetic analysis often indicated that apparent intron gains were actually numerous independent losses of the same intron. Recurrent loss exhibited extreme non-randomness, in that some introns were removed independently in many lineages. The two larger genomes, maize and sorghum, were found to have a higher rate of both recurrent loss and overall loss and/or gain than foxtail millet, rice or Brachypodium. Adjacent introns and small introns were found to be preferentially lost. Intron loss genes exhibited a high frequency of germ line or early embryogenesis expression. In addition, flanking exon A+T-richness and intron TG/CG ratios were higher in retained introns. This last result suggests that epigenetic status, as evidenced by a loss of methylated CG dinucleotides, may play a role in the process of intron loss. This study provides the first comprehensive analysis of recurrent intron loss, makes a series of novel findings on the patterns of recurrent intron loss during the evolution of the grass family, and provides insight into the molecular mechanism(s) underlying intron loss.

## Author Summary

The spliceosomal introns are nucleotide sequences that interrupt coding regions of eukaryotic genes and are removed by RNA splicing after transcription. Recent studies have reported several examples of possible recurrent intron loss or gain, i.e., introns that are independently removed from or inserted into the identical sites more than once in an investigated phylogeny. However, the frequency, evolutionary patterns or other characteristics of recurrent intron turnover remain unknown. We provide results for the first comprehensive analysis of recurrent intron turnover within a plant family and show that recurrent intron loss represents a considerable portion of all intron losses identified and intron loss events far outnumber intron gain events. We also demonstrate that recurrent intron loss is non-random, affecting only a small number of introns that are repeatedly lost, and that different lineages show significantly different rates of intron loss. Our results suggest a possible role of DNA methylation in the process of intron loss. Moreover, this study provides strong support for the model of intron loss by reverse transcriptase mediated conversion of genes by their processed mRNA transcripts.

## Introduction

Spliceosomal introns (called introns hereafter) are noncoding DNA segments within eukaryotic genes that are removed after transcription. Although the presence of introns is one of the universal features of eukaryotes, and a large number of intron positions are highly conserved in orthologous genes across species, family and even kingdom boundaries [1], [2], intron functions and evolutionary origins continue to be a subject of much debate (see reviews in [3], [4]). The number of introns varies dramatically among organisms (see reviews in [5], [6]). Accumulating evidence suggests that the common ancestors of at least several eukaryotic supergroups were intron rich [1], [2], [7], [8] and the great interspecies difference in intron density was caused by considerably different rates of lineage-specific intron loss and/or gain [3], [5].

**Figure 1:**
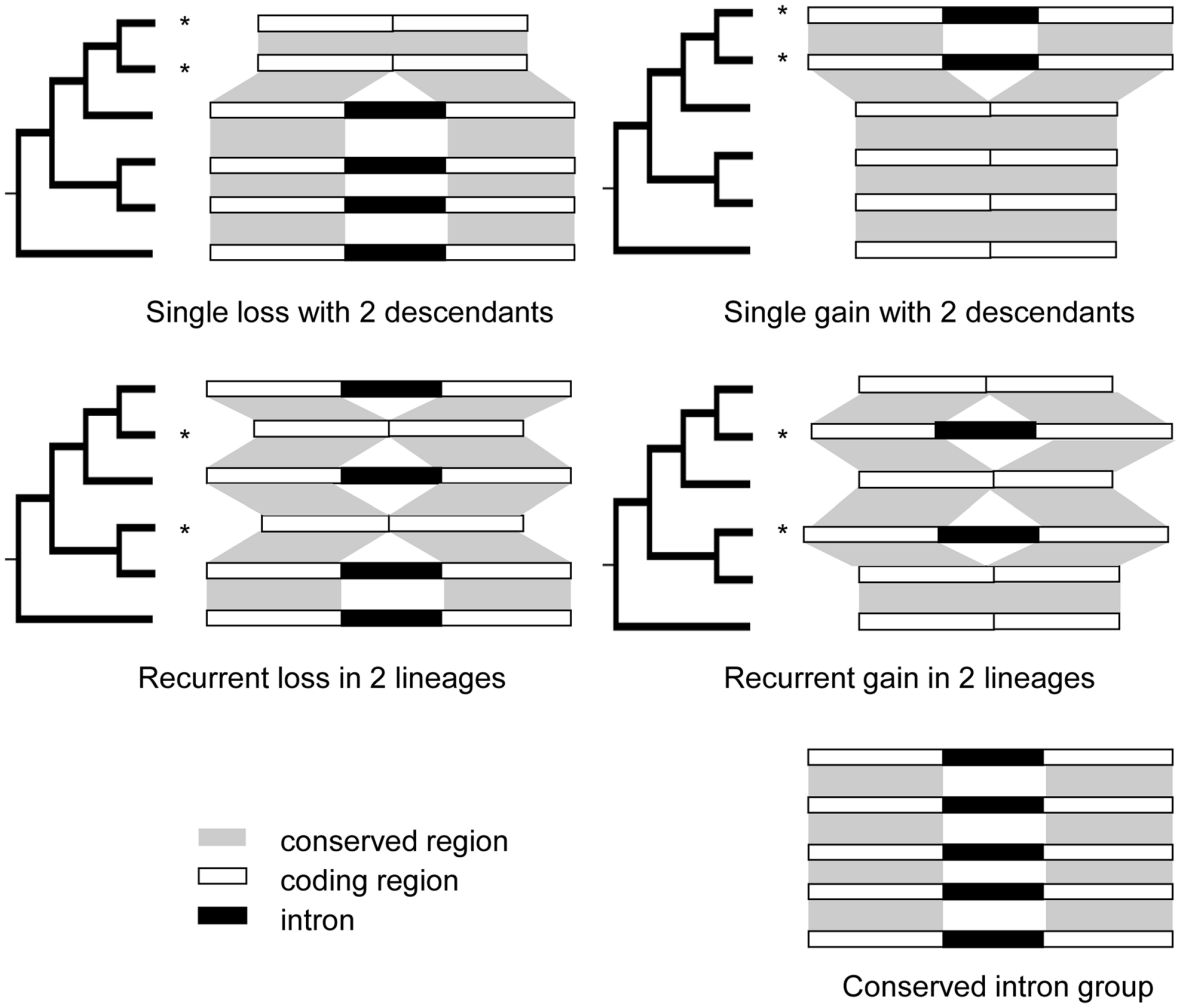
Patterns of intron loss and gain. The history of intron loss and/or gain is inferred by comparing the observed pattern of intron presence-absence with the phylogeny of the conserved genes exhibiting intron presence/absence variation using the parsimony principle. “*” denotes reconstructed loss or gain event. Bottom right example: intron exists in all genes studied, and no loss or gain has occurred. Middle left: the presence of an intron in the outgroup and two apparent losses in two lineages. Any other reconstruction requires at least 3 events. Applying the same logic, the other three patterns located at top left, top right, and middle right can be reconstructed.

Patterns of intron loss and gain have been investigated extensively in numerous subclades of the eukaryotic tree of life with different levels of taxon sampling (see review in [3]). To date, vast numbers of single loss and gain events (events inferred as occurring only once in the phylogeny investigated (Fig. 1, top) have been well-documented. Some studies also document cases of recurrent loss [9]–[11] and/or recurrent gain (otherwise called parallel gain) [12]–[14], terms describing introns that are independently removed from or inserted into the identical sites more than once in an investigated phylogeny (Fig. 1, middle).

Early examples of potential recurrent intron gain came from small scale studies of single genes, including the *Chironomus* globin gene [14] and the fruit fly and plant *xanthine dehydrogenase* (*xdh*) genes [13]. One of the reasons that recurrent intron gain has attracted particular attention is that it has been proposed as a possible explanation for the presence of introns at the same sites in orthologous genes from distant lineages under the proto-splice site model [15], [16].

Recent studies employing genome-scale data have tried to evaluate the importance of recurrent gain through broad taxon sampling [17], [18]. These studies have argued that recurrent gain is rare and that shared introns are primarily due to evolutionary conservation. In 2009, [12] reported 4 cases of recurrent intron gain in a *Daphnia pulex* population based on the most parsimonious reconstruction of intron history and supporting structural evidence, suggesting that intron gain occurs with high specificity and at a high rate in this species.

Recurrent loss of introns has been reported in the mammalian glyceraldehyde-3-phosphate dehydrogenase gene [10], dipteran *white* gene [9], and Drosophila and mosquito multidrug resistance protein *MRP1* genes [11]. Although the possibility of extensive recurrent loss in animal evolution has been proposed [4], little is known about the frequency, patterns or other characteristics of recurrent intron loss from orthologous genes since no comprehensive investigation of this phenomenon in any lineage of organisms has been reported yet.

Here we report the results of genome-wide computational identification and analysis of potential recurrent intron loss and/or gain events in five sequenced grass genomes by performing parsimonious reconstruction (Fig. 1) on trees of conserved genes and using *Arabidopsis* as the initial outgroup species (with additional outgroups used to confirm detected cases of intron presence/absence variation). The data show that recurrent intron loss accounts for at least 10% of all detected intron presence/absence variation sites. In contrast, we did not detect any clear case of recurrent gain. We further studied rate differentiation of recurrent loss in lineages, frequency of adjacent loss, position of lost introns in affected genes, expression patterns and functional enrichment of affected genes, intron size at turnover sites and their local DNA composition. The results of this comprehensive analysis yielded several observations that had previously been made in two-species comparisons, analyses of single gene families or broad multi-kingdom investigations, for instance that smaller introns were preferentially lost [10], [19], [20] and that rates of intron loss and/or gain varied between lineages [8], [21]. In addition, the observations that no recurrent intron gain events were detected, that genes with intron loss exhibited preferential expression in embryonic and/or gametophytic tissues, that lost introns had a high TG/CG ratio indicative of extensive CG methylation and that intron loss rates were similar across all chromosome regions have not been reported in any previous study of intron dynamics.

## Results

### Number and classification of presence/absence (PA) intron groups

Our intron loss and gain detection method identified 990 intron sites at which an intron was polymorphic for presence/absence in at least one of the five grass genomes (called PA intron groups hereafter; Table 1) and 24,567 introns that were present at the same sites in homologous genes in all six genomes (conserved intron groups). The PA intron groups belong to 762 OrthoMCL clusters (that is, predicted orthologous gene families) of which the number of member genes in the six genomes ranges from 3 to 141 genes. Among them, 195 clusters (containing 230 PA intron groups) had 6 member genes and 179 (212 groups) out of the 195 clusters had single copy genes in each of the six species, which gave the peak value in the cluster size histogram (Figure S1). Information on intron turnover in the 179 single-copy OrthoMCL clusters is shown in Table S1.

**Table 1:** Summary of detected intron losses and gains.

Category		Number of intron groups	Number of events	Number of affected genes(1)
Single	Gain	118	118	179
	Loss	652	652	1142
Recurrent	Gain	0	0	0
	Loss	93	206	308
	Mix(2)	20	50	169
	Unresolved	107	NA	NA
Total		990	1026	1798

(1) One gene may be counted several times since it may exhibit several categories of events.

(2) Loss followed by gain or gain followed by loss according to the most parsimonious reconstruction.

By mapping the PA intron groups onto the phylogeny of the corresponding genes and performing parsimonious reconstructions, we tried to resolve the intron loss and gain history in PA intron groups (Fig. 1 and see Methods). An intron group was called well-resolved if the minimum number of intron loss or gain events gave a unique history. To study the effects of taxon sampling on the inference of recurrent intron turnover, we added orthologous genes from banana, spikemoss, and moss, and then re-analyzed the intron loss and/or gain history in groups that appeared to have undergone recurrent intron turnover based on the analysis of the six species. We found that (1) adding these outgroups helped reduce the number of unresolved histories by providing information on the ancient states of introns in some genes (examples are shown in Figure S2). (2) Moreover, adding the banana outgroup supported the conclusion for all but 4 recurrent loss groups that the intron was present in the common ancestor of the grasses. In these 4 cases, loss of introns occurred in *Arabidopsis* as well as one or more of the grasses. For these 4 cases, adding the outgroup did not change but further supported the intron turnover history that was modeled from analysis without the outgroup. (3) In a few cases, using additional outgroups suggested a different gain/loss history than that obtained using only *Arabidopsis* as outgroup. For instance, we found 2 cases (one example is shown in Figure S3) of an apparent recurrent intron gain that was the most parsimonious reconstruction in the gene clusters composed of the five grass and *Arabidopsis* genomes alone, but adding outgroups indicated that several recurrent losses were the correct interpretation of these data.

More precision can be gained if additional outgroups are used, but this essentially infinite task would still leave some ambiguities unresolved because of haplotype extinctions. As one further test to evaluate the accuracy of our reconstructions, we downloaded grape (*Vitis vinifera*) gene annotations [22] for 50 randomly selected PA intron groups and manually checked for any change in the reconstruction of intron turnover history. In all but 1 of these cases, the intron status of grape genes fully supported our reconstructions based on the 5 grasses and *Arabidopsis* alone. In the exceptional case, the most parsimonious reconstruction as a single gain event in rice was replaced by a more likely two losses, one in *Arabidopsis* and one in the common ancestor of the grasses. This difference and re-interpretation had already been detected by the comparison to *M. acuminata*, which also contains an intron in this location.

A summary of the properties of identified PA intron groups is shown in Table 1. We found 770 groups that experienced only one loss or one gain (652 losses and 118 gains; called single event groups) and 220 groups, accounting for 22% of all PA intron groups, that required at least two events to have independently occurred. Out of these 220, 113 groups were well-resolved, including 93 groups experiencing recurrent loss (recurrent loss groups), and 20 mixed events groups (i.e., loss followed by gain (3 groups) or gain followed by loss (17 groups)).

### Intron loss is predominant

In the single event groups, the number of losses was found to be 5.5 times higher than that of intron gain (652∶118) and, in recurrent event groups, we did not detect any confirmed recurrent intron gains. When using the gene set only from the six genomes (five grasses plus *Arabidopsis*), 5 intron groups appeared to show recurrent gain. However, adding more distant outgroups indicated that 2 of them were more likely exhibiting recurrent loss and the other 3 were unresolved in the larger gene sets. In contrast, adding outgroups supported recurrent loss in most (93 out of 97) cases. In four cases, adding banana genes altered the gene tree topology and they became unresolved. Hence, recurrent gain, if it occurs at all, is much less frequent than single gain (0 vs 118 in our analysis).

### Number and orthology of recurrent loss events in groups

Because the scale of our phylogenetically analysis limited the number of recurrent events that could be detected, it was not surprising to observe that most (82 out of 93; 88%) of the recurrent loss groups experienced only 2 independent losses of any specific intron (Table S2). In fact, the maximum number of recurrent events that can be detected in the five grass genomes if an orthoMCL cluster consists entirely of five orthologous genes is 3. According to the parsimony principle, events in paralogous genes that resulted from a species-specific duplication event were counted only once. An example is shown in Figure S4, where an intron was absent in both members of this rice tandem gene pair, but only 1 loss was counted.

We found that independent intron losses occurred between orthologs in 46 recurrent loss groups and between paralogs in 41 groups, respectively. Because this analysis only captured recent paralogs due to the homology criteria involved in creating OrthoMCL clusters, these relative numbers do not provide a comparable analysis of overall intron stability in orthologs versus paralogs. In the other 6 groups, loss or gain occurred between both orthologs and paralogs. Examples of these three patterns are shown in Figure S5.

### Recurrent intron loss is not random

Overall, we detected 858 intron loss events (652 single +206 recurrent loss events, Table 1) out of a total of 176,078 intron locations analyzed which were members of 25,557 intron groups (990 PA + 4,567 conserved groups). The 858 loss events resulted in the absence of 1635 introns in affected genes. 8782 (212 PA (Table S1) +8570 conserved) out of the 25,557 intron groups had gene tree topologies identical to the topology of the species tree. In this intron group subset, the total number of intron locations was 43,910. There were 182 intron loss events (including 37 recurrent losses, Table S1) and these resulted in 257 intron losses. To determine whether some introns are preferentially lost, we randomly assigned 257 intron absences to the 43,910 intron locations and counted the number of recurrent intron loss events. This random assignment process was repeated 10,000 times and the average and maximum number of recurrent intron losses were found to be 3.5 and 19, respectively. Because the actual number of recurrent losses in this data set was 37, this indicates that the observed recurrent loss is significantly higher (p-value <0.0001) than expected based on the hypothesis that intron loss was fully random in this intron group subset.

### Lineage differentiation of events

**Figure 2:**
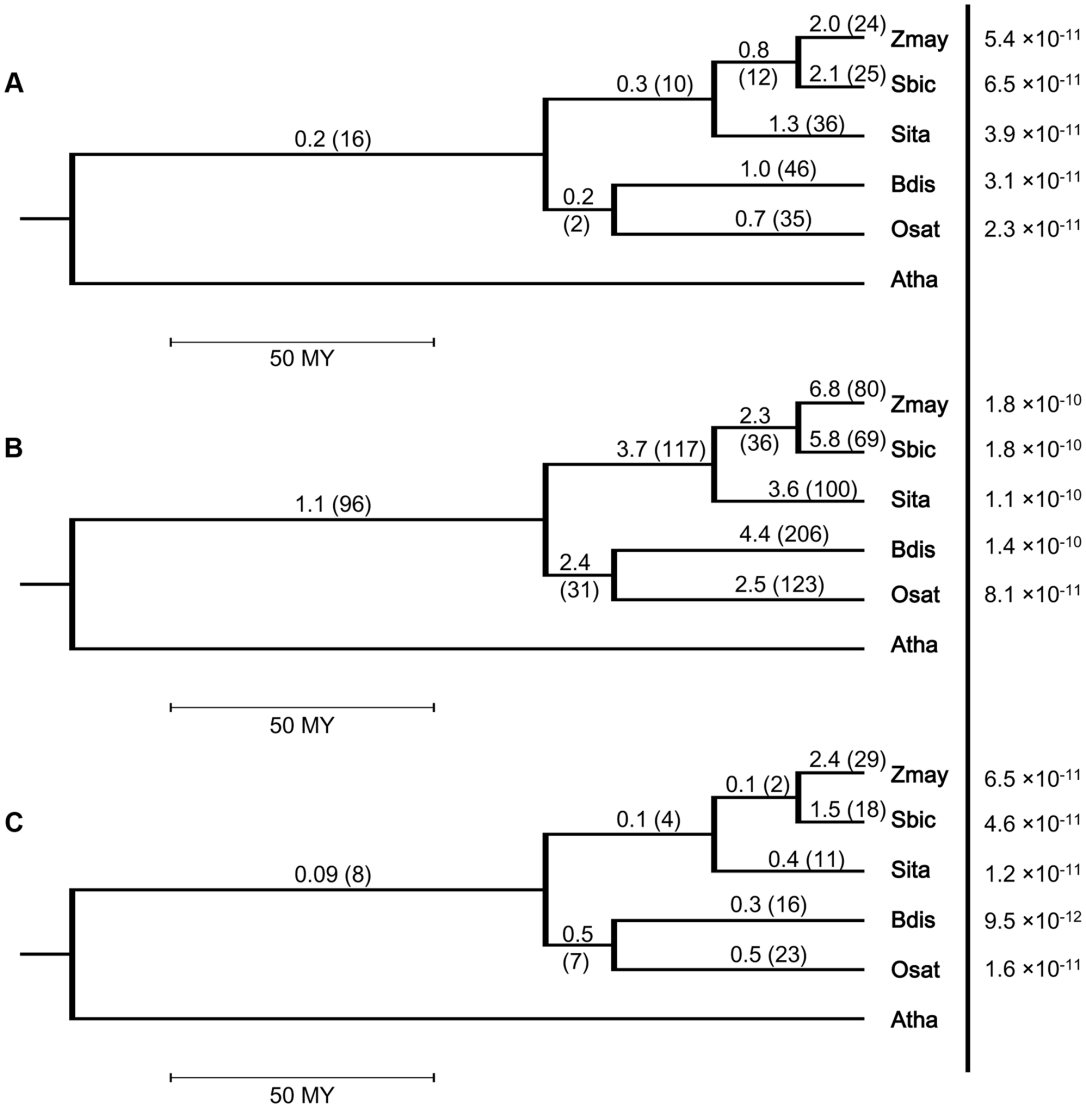
Intron turnover frequencies and rates of (a) recurrent loss, (b) all loss and (c) single gain in five grass genomes. Branches of species tree represent evolutionary time. Frequency is defined as the number of events divided by the branch length and the unit branch length is million years (MY). Rate in a terminal node is measured by the number of events per intron per year. Rates are shown to the right of the vertical line and in the same line with the corresponding species codes. The 4-letter genome codes used are Zmay: *Zea mays*; Sbic: *Sorghum bicolor*; Sita: *Setaria italica*; Bdis: *Brachypodium distachyon*; Osat: *Oryza sativa*; Atha: *Arabidopsis thaliana*.

We identified 1026 intron loss and/or gain events in the 883 resolved PA intron groups, including 879 loss and 147 gain events. The number of events in the 93 recurrent loss groups was 206, or ∼20% of all events. We investigated lineage differentiation of the average frequency of intron loss (measured by the number of events/branch length) in the grass family and our results (Fig. 2 and Figure S6) revealed that (1) single loss occurred at a higher frequency than recurrent loss in all branches (counts/time are tested; log-linear model with time as offset, p-value <0.0001); (2) sorghum and maize had significantly (counts/time are tested; log-linear model with time as offset, p-value <0.0001) higher frequency of intron loss than foxtail millet, rice or Brachypodium in both single loss and recurrent loss groups; and (3) in the BEP clade, Brachypodium had a higher (not significant, p-value  = 0.22) frequency than rice.

Another interesting trend was that the frequency of detected recurrent loss tended to be lower in more ancient branches. In the 5 terminal branches, the frequency was higher than or close to 1/MY, while in the three older branches, the Panicoid, BEP and grass values were 0.3, 0.2 and 0.2, respectively. This might be because the smaller number of ancient branches and useful outgroups are expected to yield a higher frequency of intron changes that are not resolvable at the older nodes. The lower intron loss frequency was less obvious in the single loss groups (Figure S6a), where only the branch representing the common ancestor of all grasses had a statistically significant lower (counts/time are tested; log-linear model with time as offset, p-value <0.0001) frequency than the other branches. The predicted lower intron loss frequency in ancient lineages could also be seen when calculating the frequencies for all single gain (Fig. 2c) or for all turnover events (Figure S6b).

Information on the presence or absence of a target intron in an ancestral organism can only be obtained from analyzing sister or ancestral taxa. Hence, the closer to the root of the phylogeny an event occurred, the fewer such taxa are available, thus making it more difficult to resolve more ancient events. This is expected to contribute to the lower observed frequency of intron loss observed, when compared to recent branches. Further study with increased taxon sampling will be needed to draw solid conclusions about intron loss and gain frequencies in the most ancestral stages of these lineages.

Because we know the total number of current introns that we were able to properly align, it is possible to estimate the rate of intron loss or gain events measured per intron per year in the terminal nodes. The overall number of analyzed introns in Brachypodium, rice, sorghum, foxtail millet and maize genomes were 31685, 30685, 32386, 33230 and 37119, respectively, and these numbers were used to derive the rates indicated in the terminal nodes for Fig. 2 and Figure S6.

### Intronless copies caused by retroposition or full length cDNA conversion events

Besides recurrent events, we also detected 10 OrthoMCL clusters (Table S3) that might have experienced retroposition or conversion between the intronless cDNA and the original gene copy. In their ancestral forms, the genes in these ten clusters contained at least 8 introns each. However, all introns were absent in all members of specific clades in the gene trees (Table S3). Both reverse-transcriptase-mediated (RT-mediated) intron loss (basically, cDNA-based gene conversion) [5], [23] and retroposition can lead to simultaneous loss of multiple introns, with all introns routinely being removed by the latter process. Retroposition [24], [25] usually generates an intronless copy that is located at a different locus from the original copy through the activity of retroelements like LINEs. RT-mediated intron loss, however, removes introns in the original gene but does not change its physical position in the genome. This observation provides the opportunity to discriminate between the two types of events. We found that intronless members of 2 gene clusters were located in syntenic blocks [26], [27] and thus were probably modified by cDNA conversion. Intronless members of the other 8 clusters were in new (non-syntenic) locations. In all of these cases. all introns were removed, suggesting retroposition. However, given the frequent movements of single genes to new locations that have been documented in the grasses [28]–[30], it is also possible that some of these are cases of cDNA conversion with prior or subsequent gene movement. As a control, we determined that only 33% of intron-retaining grass members of the 8 clusters were in non-sytenic blocks. Therefore, the 8/10 fully intronless genes in new locations were at a frequency suggesting that at least some lost their introns during retroposition.

### Frequencies of adjacent intron loss

In a gene, several introns may experience loss and/or gain events, and this corresponds to multiple PA intron groups in one OrthoMCL cluster. We found that 628 clusters contained 1 PA intron group and 134 contained 2 to 7 lost or gained introns. The PA intron groups at different locations exhibited identical presence/absence polymorphic patterns for introns in non-sister lineages in only 4 of these 134 clusters, suggesting recurrent loss of the same set of introns independently. In the other 130 multiple-intron-loss clusters, PA intron groups at different locations had at least two different presence/absence patterns. Furthermore, 87% of the 130 clusters contained 2 or 3 PA intron groups (Table S4) and the total number of PA intron groups in the 130 clusters was 364.

We further investigated the frequency of adjacent intron loss. We found 84 neighboring PA intron groups in the 25,557 intron groups (PA + conserved) analyzed. Among them, 57 were confirmed adjacent intron losses from the same lineage, suggesting origin in a single event. These 57 adjacent pairs belonged to 40 OrthoMCL gene clusters (Table S5). We found 11 cases of> = 3 adjacent intron losses. If adjacent triple PA intron groups (those with losses of three introns in a row in the same gene) were counted as 2 pairs, and if adjacent intron turnover events were independent, the probability of turnover of a neighbor intron could be described by a binomial distribution. A statistical test of the observed number of adjacent intron loss groups rejected the independent turnover hypothesis at the 0.05 level.

In cases where adjacent introns were lost from the same lineage, a size-limited model of intron loss (for instance, by nuclear cDNA conversion or aberrant double-strand break repair) would suggest that adjacent introns with a smaller intervening exon would be more likely to be lost in a single event. Surprisingly, we found that the sizes of intervening exons was not significantly different between adjacent introns lost in a single lineage (presumed single events) when compared to the intervening exon sizes of adjacent introns lost in two independent events (Figure S7).

### Intron turnover location in a gene

When intron turnover occurred in a terminal node of the gene tree, the gene that underwent intron loss and/or gain was the affected gene of that event. When intron turnover occurred in an internal node, all descendants of that node were taken as affected genes. Therefore, one event might have more than one affected gene. The total number of genes that underwent intron turnover was 1798, including 308 involved in the category of recurrent loss, and 1142, 179, and 169 in single loss, single gain and gain/loss mixtures, respectively (Table 1). Some genes were counted more than once because intron turnovers occurred at multiple locations in some gene clusters. For example, if two introns in a gene family experienced recurrent loss and single loss, respectively, this gene was counted twice. If all genes were counted only once, the total number of affected genes of intron turnover was 1720.

**Figure 3:**
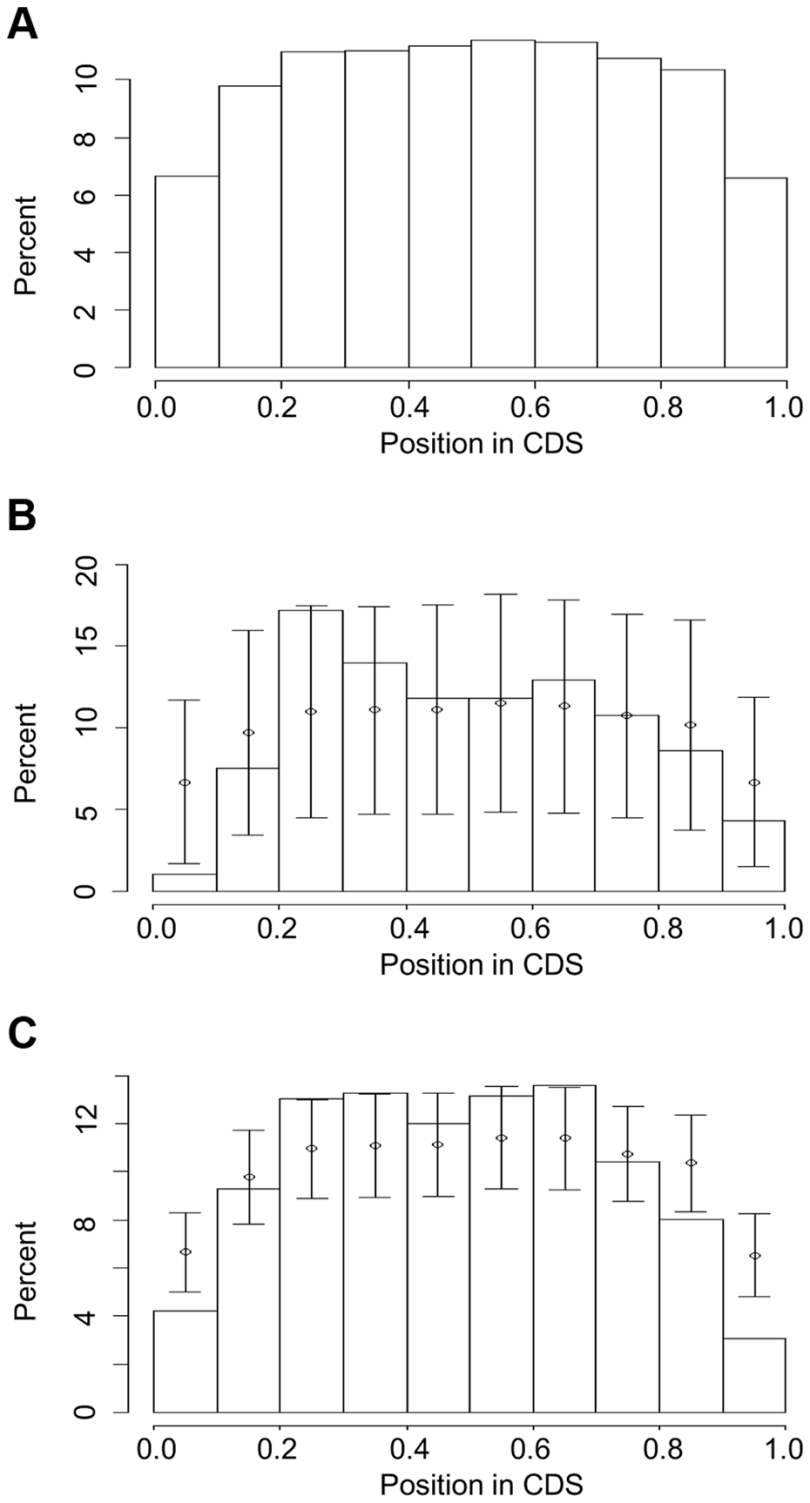
Intron locations in genes from five grass genomes: (a) all introns, (b) recurrent loss introns and (c) PA introns. The CDS lengths in genes are normalized to 1 and the positions of introns are calculated as (length from translation start)/(total size of CDS). The normalized gene is partitioned into 10 intervals (X-axis) and Y-axis values are the percentage of introns in these intervals. Error bars in (b) and (c) represent one sd from interval mean values (circle), where mean and sd are calculated by resampling with replacement (1000 times) from the entire intron set.

We normalized for gene size and investigated the distribution of intron loss or gain along each gene (Fig. 3 and Figure S8). The locations of all introns in all gene models of the six genomes showed a relatively even distribution, with the two termini, i.e. (0, 0.1) and (0.9, 1.0) of the total length of a gene when calculating from the 5′ end, exhibiting lower values (Fig. 3a). Under-representation of intron loss and/or gain at the gene termini was also observed in recurrent (Fig. 3b) and PA intron groups (Fig, 3c): the percentage of recurrently lost and PA introns located at (0, 0.1) and PA introns at (0.9, 1.0) was lower than expected based on the mean and sd from 1000 resamplings from all introns. In the all loss (recurrent + single) groups, we observed (0.2, 0.3) had a percentage higher than expected based on the resampling results (Figure S8).

### Chromosomal distribution of genes exhibiting intron turnover

**Figure 4:**
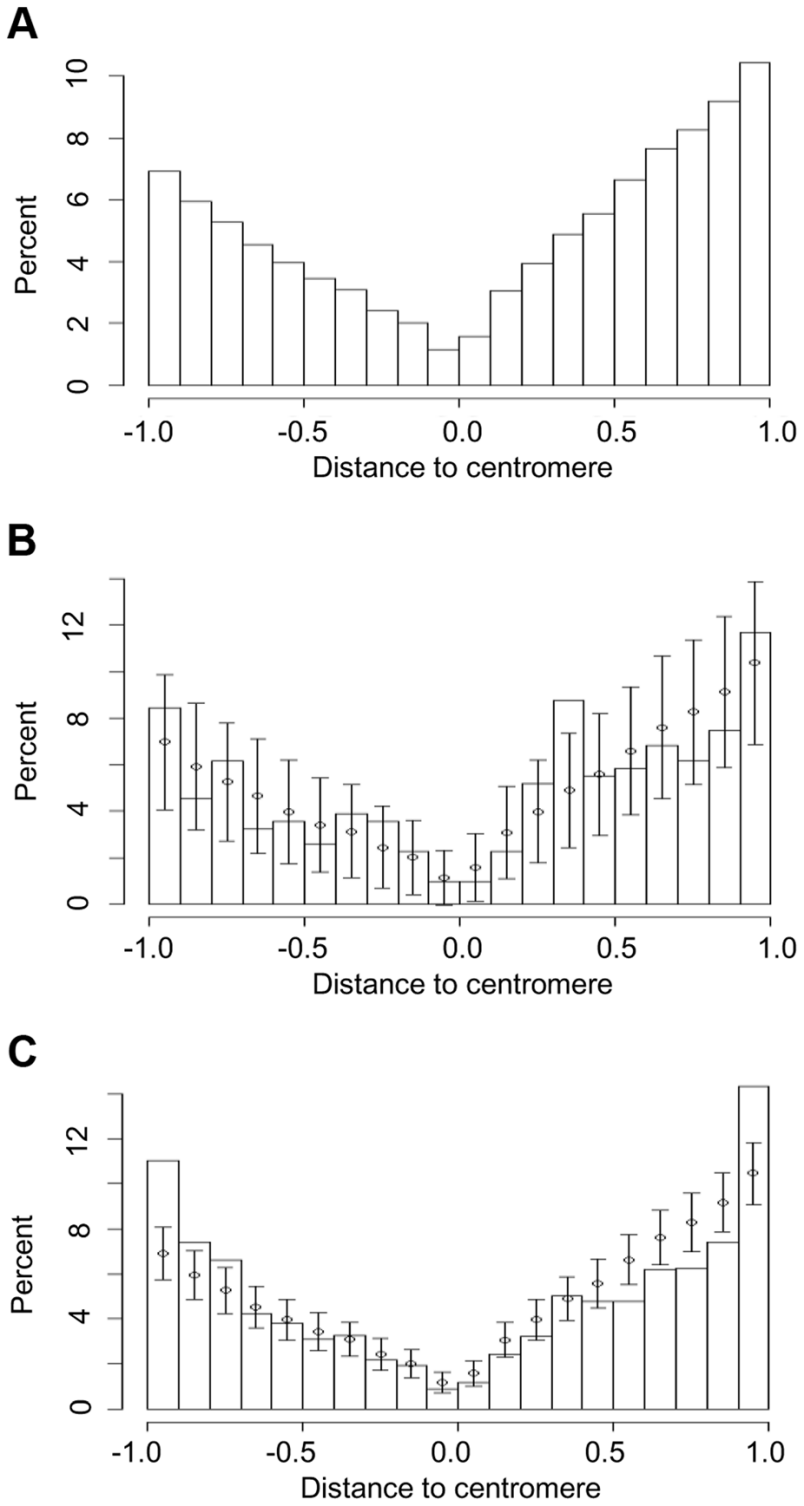
Chromosomal locations of (a) all genes in five grass genomes and those that have undergone (b) recurrent intron loss or (c) any PA intron variation (gain or loss). Short arms and long arms of chromosomes are normalized separately. The centromere is located at 0 and the short and long arm termini at -1 and 1, respectively. The locations of genes are calculated as distance from centromere divided by total length of chromosome arm, where distance is a negative number for the short arm. The normalized chromosome is partitioned into 20 intervals (X-axis) and Y-axis values are the percentage of genes in these intervals. Error bars in (b) and (c) represent one sd from interval mean values (circle), where mean and sd are calculated by resampling with replacement (1000 times) from the entire intron set.

We investigated the distribution of intron turnover across chromosomes (Fig. 4 and Figure S9) by normalization of chromosome size. The centromere was located at 0; and then the short arms and long arms of chromosomes were normalized separately, with the short and long arm termini located at -1 and 1, respectively. Locations of genes belonging to the same intron group were counted independently. The distribution of the whole gene set of the five genomes (Fig. 4a) showed a smooth “V” shape with the lowest gene density located at the centromeric/pericentromeric region. The distributions of genes with detected recurrent loss (Fig. 4b) and total intron turnover (Fig. 4c) exhibited a similar overall trend. However, it should be noted that plant genes are highly mobile over evolutionary time, and even centromeres can be found in different positions in closely related lineages [31], so the current genomic location is not a perfect predictor for any single gene of its location when an intron was gained or lost.

Resampling showed that the percentage of genes that underwent recurrent intron loss located in (0.3, 0.4), the region close to the middle of a normalized long arm, was higher than expected from the resampling mean and sd (Fig. 4b). The density ratio calculated as the density (number of genes per normalized unit length of a chromosome) of genes with recurrent intron loss divided by the density of the entire gene set exhibited a peak at this region (Figure S10), suggesting hotspots for intron recurrent loss events at these regions. When considering all PA introns, a higher than expected percentage of affected genes was located at the chromosome arm ends (−1, −0.9) and (0.9, 1) (Fig. 4c).

### Frequency of intron turnover in genes with single and multiple introns

We found that 698 (91.6%) of the 762 OrthoMCL clusters with intron turnover events were derived from genes with multiple introns and the other 64 (8.4%) were from ancestral genes with a single intron. The 93 resolved recurrent loss intron groups belonged to 88 gene clusters, 4 (4.5%) of which were from single-intron ancestral genes. The frequency of all loss and recurrent loss occurring in single intron genes is 8.4% and 4.5%, respectively. A Pearson's Chi-square test (p-value  = 0.2918) indicated that, compared to the PA intron groups, the number of single-intron genes was neither over-represented nor under-represented in the recurrent loss groups.

A total of 1026 confirmed intron turnover events (Table 1) were found among all introns analyzed in the grasses (588,669) and 65 of these events were in one of the 18,226 single-intron genes. A Pearson's Chi-square test (p-value  = 1.75e-8) indicated that the rate of intron turnover in single intron genes (65/18,226 or 0.36%) is significantly higher than the rate of intron turnover overall (1026/588,669 or 0.17%).

### Phase distribution of intron turnover

We investigated the codon phase distribution of intron turnover events and found that 58% (575/990 PA intron groups), 20% (195) and 22% (220) of turnover events involved introns in phase 0 (intron-exon boundaries located between two codons), phase 1 (between the first and second base of a codon), and phase 2 (between the second and third base of a codon), respectively. The excess of phase 0 introns has been well-documented (see review in [3]) and intron phase distribution in rice has been estimated at 57∶22∶21 for phase0: phase1: phase2 [32], very close to our estimation for intron turnover in the five grass genomes (56∶21∶23). Statistical analysis (Pearson's Chi-square) indicated no significant difference between the overall intron phase and PA intron phase data.

### Expression patterns of genes that underwent intron loss and/or gain

We investigated the expression patterns of the 289 rice genes that exhibited intron turnover because publicly available expression data are relatively abundant for rice. A total of 283 out of the 289 genes matched probes in the rice 57K Affymetrix GeneChip (http://www.affymetrix.com/). 283 probes were selected through PLExdb's “Gene List Suite” tool using these genes as queries. Each probe corresponded to a single gene. When a gene matched multiple GeneChip probes, the probe with highest BLAST bit score was used to represent the gene. We extracted gene expression information for eleven stages in early embryogenesis and six for pollen development from multiple experiments deposited in PLEXdb (Table S6; see Methods). Since expression values are not comparable between different experiments, we only focused on whether genes that underwent intron turnover were detectably expressed or not in a given tissue. As shown in Table 2, compared to total nuclear genes, significantly higher (Pearson's Chi-square test, see Table 2) percentages of genes with intron loss events were expressed in both early embryogenesis and pollen development both when considering the average percentage of genes expressed in all developmental stages and the percentage of genes expressed in at least one of the developmental stages. Genes in the PA category, which includes all genes that underwent intron loss and/or gain, also were expressed significantly more frequently in these two expression categories than total nuclear genes. The fact that genes that have undergone intron loss exhibit a higher frequency of germ line or early embryogenesis expression than observed for the complete transcriptome suggests that expression in heritable tissues is associated with transmission of the intron removal outcome.

**Table 2:** Expression pattern of host genes of intron groups.

Gene category	Number of genes	% expressed genes in early embryogenesis(1)				% expressed genes in pollen development(2)			
		Mean(3)	P-value(4)	At least one stage(5)	P-value(4)	Mean(3)	P-value(4)	At least one stage(5)	P-value(4)
Recurrent loss	52	53	0.25	81	0.035	23	0.63	50	1
Loss	207	68	4.5E-11	89	6.4E-12	36	2.2E-16	64	3.2E-4
PA	283	67	1.3E-13	88	1.1E-14	34	0.011	63	8.3E-5
Whole genome	57,381	45	NA	66	NA	27	NA	51	NA

(1) Eleven developmental stages are analyzed. Details are show in Table S6.

(2) Six developmental stages are analyzed. Details are show in Table S6.

(3) Average percentage of genes expressed in all developmental stages investigated.

(4) P-values of Pearson's chi-square test, comparison to the Whole genome category.

(5) Percentage of genes expressed in at least one of the developmental stages investigated.

GO analysis of genes in all intron gain and/or loss categories indicated numerous terms that were over-represented at a level considered statistically significant by the GO analysis software (see Methods). These over-represented categories are shown in Figure S11. Among them, the top five most significant GO terms were from the following functional categories: ‘catalytic activity’, ‘oxidoreductase activity’, ‘metabolic process’, ‘omega-3 fatty acid desaturase activity’ and ‘positive regulation of protein modification process’.

### Intron size

We compared the size and composition of PA intron groups to that of all intron groups. The average sizes of introns in recurrent loss intron groups, all PA groups and conserved groups were 212 bp, 266 bp and 360 bp, respectively. The average size of PA introns (total intron count  = 7950) and recurrent loss introns (1097) were both significantly shorter than that of conserved introns (201,660) (Mann-Whitney test, p-value <2.2e-16). The preference for loss of short introns has been reported previously [10], [19], [20]. It is not clear why smaller introns might be more easily lost, although two distinct possibilities come to mind. Smaller introns may be less likely to contain important regulatory modules, so that their loss would be less likely to detrimentally affect gene function. A second possible explanation is that intron removal (perhaps by a cDNA conversion process or aberrant NHEJ repair) is tolerated only if complete because partial removal would leave an unspliceable intron fragment, and thus smaller introns would be more likely to be fully removed in any time-constrained process. A third possibility is selection for smaller transcript size or fewer introns so that genes might be more rapidly transcribed and/or matured to increase gene expression [33]. However, we observed that small genes were just as likely to lose and/or recurrently lose introns as large genes (Figure S12), indicating that transcription rate is not a clear factor in any possible selection for intron loss. We also did not observe any correlation between organismal genome size and overall intron number or intron loss rate (Figs. 2, Figure S6), nor exceptionally frequent loss of introns from genes with large intron numbers (Figure S13), so a model suggesting selection against introns per se is not supported by these observations. The observation in Figure S13 that introns are most frequently lost from genes with few introns does not provide any obvious mechanistic model for this preferential loss, but does suggest that many of these genes with very few introns are the ongoing products of especially frequent intron loss. Consistent to our observation of non-random loss of introns, Figure S13 suggests that not all introns in a gene have an equal likelihood of being removed.

Although recurrently lost introns, like single-loss introns, are smaller in size than the average intron, this fact does not by itself explain recurrent loss. That is, if one compares the size of recurrently lost introns, there is no significant different in their size compared to single loss introns (Figure S14).

### DNA composition

We investigated the nucleotide compositions of different categories of intron groups (recurrent loss, PA, and conserved intron groups) as well as their flanking exonic sequences in the five grass genomes. As depicted in Table S7, the mean G+C content of recurrent loss introns, PA introns and conserved introns identified in OrthoMCL groups were very similar (39%) and lower than the genome-wide G+C content (44%) or the average G+C content of exons (55%). The lower G+C content of introns compared to exons in plants is a well-known phenomenon (e.g. [34]–[36]) and is believed to be related to the high G+C content of plant triplet codons [37], [38]. The mean G+C contents of the last 20 bp of the upstream flanking exon and the first 20 bp of the downstream flanking exon were similar in recurrent loss and PA intron categories (55%). This value is identical to the genome-wide G+C average for exons. Interestingly, the mean G+C content of the 20 bp of exonic sequences flanking conserved introns (47%) was about 8% less than that of the 20 bp of exonic sequences flanking PA introns. Pearson's Chi-square test indicates that this difference is highly significant (p-value <2.2e-16).

We investigated dinucleotide frequencies for all PA and conserved introns. The three intron categories (recurrent loss, PA and conserved) exhibited similar frequencies of different dinucleotides (Table S8). In all three categories, TT, AT, AA, TG and TA were the most abundant dinucleotides, while GC, CC, GG and CG were the least abundant dinucleotides. A similar tendency was found in the genome-wide dinucleotide frequency, where CG had the lowest frequency and AA, TT and AT had the highest frequencies. While CG was also the least frequent dinucleotide in exons flanking conserved introns, it was TA that had the lowest frequency in exons flanking lost introns, in agreement with the higher AT-richness of exons flanking conserved introns mentioned above. Within introns, TG was the fifth (recurrent loss), fourth (PA) and third (conserved) most abundant dinucleotide across the three different categories. Interestingly, the TG/CG ratio was 2.4, 2.9 and 4.2 in recurrent loss, PA and conserved categories (Tables S9 and S10). The differences in the TG/CG ratios among the three intron categories were highly significant in terms of Pearson's Chi-square test with Bonferroni correction (Table S10). A low “CG” and high “TG” frequency suggests the process of “C” to “T” transition that is enhanced by 5-methylation at cytosine bases [39], [40]. Introns in general are observed to be relatively less methylated than exons in plants, especially at cytosine bases [41]. This result suggests that introns with a history of less CG methylation are more likely to be removed.

Flanking exons of conserved introns also had a relatively higher TG/CG ratio than flanking exons of PA and recurrent loss introns (TG/CG = 2.0 *vs*. 0.9 and 0.77; Table S9). Hence, these results suggest that conserved introns and their flanking exons were relatively highly methylated while intron turnover tended to occur where the degree of cytosine-5 methylation in both the flanking exons and introns was relatively low.

### Intron-exon junction sequences associated with intron PA variation

Although the GT….AG terminal intron dinucleotides are most abundant in all studied eukaryotes, including plants, rarer junction sequences are also found [42]. Table S11 shows the terminal intron dinucleotides associated with conserved introns and PA introns. No significant differences were observed between conserved or PA introns, regardless of whether the intron location had the added precision of confirmation by transcript analysis.

## Discussion

### Frequency of intron loss is far higher than gain

Many previous parsimonious reconstructions of intron loss and/or gain [2], [43] were based on Dollo parsimony, which assumes that every intron arose only once along the tree and thus explicitly excludes parallel gains. Recent studies, however, suggest that this assumption is not valid [8]. Prohibiting recurrent intron gain is also a characteristic of some probabilistic methods [44]–[46]. However, one of our aims was to provide an unbiased assessment of the frequency of intron gain. Hence, a cladistic parsimonious counting strategy (See Fig. 1 and Methods) was employed that initially modeled the fewest intron turnover events in the tree but did not restrict the occurrence of recurrent gain or loss. The parsimony method was valid as a first step in the analysis as we had no prior knowledge on the frequency of gain or loss. Our results indicate that intron loss is much more frequent than intron gain (858 losses (206 recurrent events +652 single events): 118 gains  = 7.2: 1) (Table 1), consistent with results from several previous investigations in plants [32], [47], [48].

Use of a simple cladistic approach to predict the origin of a rearrangement is not valid when the rates of the two mechanisms of rearrangement are quite different. In our case, for example, where intron gain occurs at a 7-fold lower rate than intron loss, parsimony tends to overestimate gain and underestimate loss since it cannot be guaranteed that any of the events judged as intron gains are not actually cases of multiple independent losses that could not be resolved due to a lack of phylogenetic power. In fact, in all unresolved and mixed groups, the gain events predicted by the parsimony principle could also be explained by two or more recurrent intron losses. In 18% (21 out of 118) of single gain groups, the gain can be substituted by 2 losses. If every gain is allowed to be substituted by at most 3 losses, the percentage reaches 31% (37 out of 118). Given the at least 7.2 fold higher frequency of losses than gains that we observed under the parsimony model, even models with 3, 4, 5 or 6 recurrent losses are more likely than single gain events, thus allowing all of the gains to be potentially explained by recurrent loss.

### Proposed mechanisms suggest a low rate of intron gain

The origin of new introns requires the insertion of precisely positioned functional splicing signals inside (and, presumably by chance, flanking) the new intron. Unlikely as this seems, many models (see [49] for a short summary) of frequent intron gain have been proposed, including (1) modification of self-splicing introns [50]; (2) intronization of coding sequences [51], [52]; (3) transposon insertions that become precise introns [53]–[55]; (4) tandem duplication of coding sequence with an AGGT motif to create an intron terminal signal [50]; (5) nonhomologous end-joining (NHEJ) of DNA segments [12], [56]; and (6) intron transposition [57]–[59]. In plants, only one of these models, a transposon insertion turned intron, has been supported by *in vivo* evidence [53]–[55]. One case has been observed in which a new intron did not dramatically debilitate gene function because it was precisely processed [55]. However, none of the proposed intron gains that have been fixed in a species have been associated with a transposable element insertion/structure, so rare intraspecies variation for this trait may be more an indication of mutational neutrality than a major mechanism of evolutionary change by intron gain.

### Does recurrent gain ever occur?

The rarity of intron gain implies that the chance of recurrent gain should be even lower. Previous reports of recurrent intron gains [12]–[14] were based exclusively on a cladistic approach with few investigated taxa. The main reason that these investigators preferred recurrent gains is that the intron distribution in their phylogeny could be explained more parsimoniously by invoking less independent intron gains than losses: 2 gains v.s. 8 losses in the globin gene analyzed by [14] and 2 gains v.s. 14 losses in the *xdh* genes analyzed by [13]. As mentioned above, when the frequency of loss is much higher than gain — and this has been supported by the current study as well as by previous analyses [10], [32], [47], [48] — the cladistic approach tends to mistake recurrent losses as individual or recurrent gains. Furthermore, as is shown in our study, using a single species as outgroup may lead to incorrect assumptions on the ancestral state of an intron. In short, more convincing evidence (such as that provided in [12]) of recurrent intron gain is needed to determine whether this proposed phenomenon has a solid foundation in plants. However, phylogenetically deep and/or molecularly detailed studies of intron gain have recently been published for a number of organisms [12], [56], [60]–[64], so it will be interesting to see if any future plant studies uncover similar phenomena.

### Recurrent loss may be going on at a higher frequency than reported

Including outgroups, the phylogenetic scope of the three previously reported cases of recurrent intron loss were in single genes in vertebrates [10], Bilateria [9] or Pancrustacea [11]. In our research, the scope is extended to the last common ancestor of monocots and dicots. During the ∼150 MY evolution of the grass family since their divergence from a common ancestor with *Arabidopsis*, we have identified 93 (initial parsimony result) to 200 (all possible cases, including those currently unresolved) polymorphic intron sites that are likely to have experienced recurrent losses. The rate of recurrent intron loss thus is 2.4−5.2×10^−5^/MY/intron site (total analyzed intron sites  = 25,535). The counting of recurrent loss is affected by the depth of phylogeny and taxon sampling. For example, recurrent losses in rice and maize will appear as a single loss in each species if BEP clade and PACCAD clade species were investigated separately. Here BEP is an abbreviation representing the clade of grass subfamilies Bambusoideae, Ehrhartoideae, and Pooideae; and PACCAD an abbreviation representing the clade of grass subfamilies Panicoideae, Arundinoideae, Chloridoideae, Centothecoideae, Aristidoideae and Danthonioideae. As a further investigaton of this point, we extended the intron loss analysis for six sets of orthologous genes that contained a total of 9 recurrent loss groups and 18 intron loss events by manually aligning the orthologs from the five grass species. These were analyzed with the orthologs from one additional species (2 genes) or two additional species (4 genes) for which sequence information was available, and this identified 3 additional recurrent loss events, all in the same gene (unpub. obs). Thus, adding more taxa will lead to the detection of more recurrent losses, indicating that the current estimates are very conservative minima.

### Can selection explain recurrent loss?

Our results show that recurrent loss accounts for a considerable proportion of all detected intron losses. Moreover, loss is not random, but involves a small subset of introns that are lost over and over again in multiple lineages. Our analyses indicate that some of these introns may have been lost repeatedly because of their (1) small size, (2) expression in tissues that contribute to the germ line or (3) history of less 5-methylation in regional CG dinucleotides. Fawcett and colleagues [48] suggested that the high rate of intron loss in a small genome might be caused by strong selection for genome reduction in their study of two *Arabidopsis* species. However, consistent with previous studies [10], [19], [20], our results shows that short introns are preferentially lost, a result not consistent with selection for intron loss as a mode of genome size reduction. Moreover, we observed a higher frequency of intron loss in larger compared to smaller grass genomes. Both observations suggest that selection is not on the basis of an effect on genome size. Given that the loss of a single intron in plants will only decrease genome size by a few hundred bp in most cases, it is difficult to see how this would be detected and/or significant within a several gigabase genome. Another theory is that loss of an intron could lead to more efficient transcription [33], but this model is also inconsistent with a preference for short intron removal.

Based on our observation that lowly methylated introns are more likely to be removed than highly methylated introns, it is possible that there might be selection related to the epigenetic status of genes. Methylation of DNA is associated with specific chromatin compositions/conformations and an epigenetic status that tends to have negative effects on transcription [65], [66]. Hence, loss of an intron with a particular level of DNA methylation might be expected to have a selected epigenetic outcome, perhaps leading to an altered level or timing of gene expression.

### Mechanisms that drive intron loss

Although natural selection might explain some or all recurrent losses, it is also possible that coincidental features of intron structure, gene location and/or gene expression might explain a high rate of removal for specific introns. Two general mechanisms for intron loss have been proposed, but neither has gained comprehensive support yet [49]. One proposed model is intron loss by genomic deletion [4], [5] via NHEJ [48] or other molecular mechanisms, leading to exact [48], [67] or inexact [20], [68] intron removal. The other proposed mechanism is an RT-mediated intron loss model. The standard version of this mechanism predicts that introns located at 3′ ends of genes are more likely to be lost because reverse transcriptase reads/polymerizes from 3′ to 5′ along the RNA template and often produces incomplete transcripts [5], [69], [70]. However, a lack of 3′ bias of intron loss has been reported in several species, including plants [32], [48], animals [43], [71] and fungi [46], [60]. So, modified RT-mediated intron loss models involving self-primed reverse transcription have been proposed [72]–[74], but there is not yet any compelling evidence to support these models [43], [60], [75].

Our data indicated that large grass genomes (sorghum and maize) have a significantly higher intron removal rate and that short introns are more commonly removed. These observations are compatible with the hypothesis that RT-mediated intron loss plays a role in driving intron loss because large genomes contain more class I TEs and thus may have higher reverse transcriptase activity. Furthermore, smaller introns are more likely to be lost by RT if the enzyme is prone to incomplete template coverage. In addition, several other results in this study provide indirect support for an RT-mediated intron loss mechanism, including (1) adjacent intron loss occurring at higher frequency than expected by chance, (2) genes exhibiting intron loss are enriched in germline and early embryogenesis transcriptomes; and (3) deletions of introns are exact. The lack of a 3′ bias for intron loss detected in our study, however, conflicts with the simplest models of cDNA conversion for intron loss, as does the observation that the concurrent loss of adjacent introns does not seem to be highly affected by intervening exon size. Another model, involving ncRNAs that direct DNA rearrangement (including deletion) [76], does not necessarily require RT activity or 3′-end priming at an mRNA polyA, but still would predict a more frequent loss of adjacent introns if they were separated by smaller exons. To increase our understanding of these issues, studies are needed to investigate *de novo* intron loss, and these studies would be best performed with introns in genes that exhibit a history of recurrent loss in other lineages and in genetic backgrounds that are enriched for RT and/or for NHEJ activities.

## Materials and Methods

### Genomic sequences and centromere positions

The genomic sequences and annotation data for maize (*Zea mays*, version 5b.50) and rice (*Oryza sativa*, IRGSP/RAP Build5) were downloaded from MaizeSequence (http://www.maizesequence.org/index.html), IRGSP (http://rgp.dna.affrc.go.jp/E/IRGSP/) and RAP-DB (http://rapdb.dna.affrc.go.jp/) databases. Data for the sorghum (*Sorghum bicolor*, version 1.4), foxtail millet (*Setaria italica*, JGI release 164) and Brachypodium (*Brachypodium distachyon*, JGI release 114) genomes were obtained from the Phytozome website at DOE-JGI (http://www.phytozome.net/). Data for *Arabidopsis thaliana* (version TAIR10) were retrieved from TAIR (http://www.arabidopsis.org/). Raw reads for the five grass genomes were downloaded from the NCBI Trace Archive Database (ftp://ftp.ncbi.nih.gov/pub/TraceDB/) and the reads for *Arabidopsis thaliana* were from the 1001 Genomes project website (http://1001genomes.org/). Besides these six species, sequences and annotation of banana (*Musa acuminata*, version 1), was downloaded from The Banana Genome Hub (http://banana-genome.cirad.fr/); data of spike moss (*Selaginella moellendorffii*, JGI release 91) and moss (*Physcomitrella patens*, JGI release 152) were also downloaded from Phytozome v8.0.

The estimated positions of maize, sorghum, foxtail millet, Brachypodium and Arabidopsis centromeres were directly extracted from several earlier publications [77]–[81]. The positions of rice centromeres were collected from the Rice Genome Annotation Project (http://rice.plantbiology.msu.edu/annotation_pseudo_centromeres.shtml), and sequences around these regions were compared to IRGSP/RAP Build5 to find the corresponding locations.

### Detection of intron loss and gain events

Intron group identification: The workflow of intron group identification is shown in Figure S15. Firstly, the representative gene repertoires of the five grasses and *Arabidopsis* were extracted and orthologous clusters (called OrthoMCL clusters) were built using OrthoMCL [82] with default parameter settings. Here, we used the longest transcript as a representative sequence for each gene. Secondly, protein alignment-guided multiple sequence alignments (MSA) of coding DNA sequences (CDS) of each OrthoMCL cluster were constructed using TranslatorX [83] with default parameter settings. Next, OrthoMCL clusters too large or small (>200 or <3 member genes) or clusters of which>30% of the members matched known transposon proteins were excluded. This last step was designed to remove from consideration those gene models where transposable elements (TEs) were found inside coding sequence, because we have noted that most of these are mis-annotations (especially pseudogenes mis-annotated as genes) ([84], H. Wang and J. Bennetzen, unpub. res.). The analyzed genes that remained included many cases of introns (both conserved and PA variants) that contained TEs.

Subsequently, we extracted intron positions from gene models annotated in the various genomes and mapped these positions to the above protein-guided CDS alignment. An intron in the genomic sequence thus was mapped to a position between two consecutive bases corresponding to the last base of exon k (base i) and the first base of exon k+1 (base i+1) in a CDS (Figure S15, top right). In the CDS alignment, whenever a position between two consecutive bases had break points, we called this position an intron group. If all member genes in the intron groups had break points, this indicated that each member gene had an intron at the same position and this group was judged a conserved intron group. If only some member genes had break points, the group was called a PA intron group (Figure S15, middle left). We identified all intron group candidates for all OrthoMCL clusters with this method. We further required that the up- and downstream exons of detected intron groups were well-aligned (flanking exons in each member gene exhibited ≥60% identity to consensus sequences of the flanking exon alignments) (Figure S15, middle right). This homology restriction led to the selection of conserved intron sites and excluded artificial intron groups caused by MSA algorithms. One possible issue was “intron sliding” [3], [85], where an intron was not actually lost or gained, but moved one to several nucleotides away due to a shift in the intron/exon boundaries. With an inappropriate detection method, “intron sliding” might be perceived as an intron gain, intron loss or (most likely) reciprocal intron gain and loss in two different lineages. Hence, we required that the alignment be perfect at the exact ends of the intron/exon junction, and that no two intron PAs were allowed to be within <20 bp of each other. We manually checked all 990 of our PA intron groups to see if any were due to intron sliding, and none were. Next, we excluded intron groups containing very short introns (≤10 bp) to avoid artifacts generated by incorrect intron annotation (Figure S15, bottom). Finally, selected intron groups were compared with raw reads to confirm that the presence or absence of introns in the groups was not caused by assembly errors (Figure S15, bottom and Figure S16).

Another possible issue with these analyses involves the quality of the gene annotations that we accepted from the published genomes investigated. In particular, it was not clear whether we would see any differences in intron presence/absence variation properties in cases where introns were confirmed by transcript analysis. Transcript data (e.g. ESTs) covered the breakpoint plus at least 20 bp upstream and downstream of the intron boundary for 946 of the 990 PA intron groups in at least one of the species investigated. Although the number of PA introns without transcript support was too small to allow statistically significant values to be obtained when comparing PA intron properties to those PA introns with transcript support, in all cases the trends were in the same direction.

Gain or loss event resolutions: For every PA intron group, we mapped the intron presence/absence pattern on the corresponding gene tree. The history of intron turnover of the group was reconstructed according to the parsimony principle which assumes that the history with the lowest number of intron turnover events has the highest likelihood of representing the true chain of events (Fig. 1). If the parsimonious reconstruction corresponded to more than one possible intron loss and/or gain history, the intron group was called an unresolved group. For every group for which recurrent events were inferred from the six genome analysis, we added in orthologous genes from banana, spike moss and moss and redid the intron loss and/or gain history inference. This analysis allowed us to confirm reconstructions based on fewer species and demonstrate that none of the initial recurrent gains calls were supported by the broader cladistics analysis. Detailed information for all intron loss events identified in this study is provided in Table S12.

### Gene tree construction

TreeBeST (http://treesoft.sourceforge.net/treebest.shtml) was used to build the gene trees for the OrthoMCL clusters. The program constructed trees with the Maximum Likelihood method under guidance of the species tree. The species tree topology used in this study was (((((((Zmay, Sbic)Andropogoneae, Sita)Panicoid,(Bdis, Osat)BEP)Grass, Muca)Monocot, Atha)Angiosperm, Smoe), Ppat). The 4-letter genome codes used were Zmay: *Zea mays*; Sbic: *Sorghum bicolor*; Sita: *Setaria italica*; Bdis: *Brachypodium distachyon*; Osat: *Oryza sativa*; Muca: *Musa acuminata*; Atha: *Arabidopsis thaliana*; Smoe: *Selaginella moellendorffii*; Ppat: *Physcomitrella patens*.

### Rates of occurrence of intron loss or gain

Using previous estimations of the divergence time of the five grass species, i.e. 150 million years (MY) between *Arabidopsis* and grasses [86], [87]; 60 MY between BEP and Panicoids [87], [88], 47 MY between rice and *Brachypodium* [77], 26 MY between foxtail millet and Andropogoneae [78], and 12 MY between sorghum and maize [89], branch lengths of the grass species tree could be scaled as evolutionary time. The mean rates of intron loss or gain in branches were calculated as the number of events divided by the branch length.

### Gene ontology analysis

GO annotation and enrichment analysis of genes exhibiting intron loss or gain were performed in AgriGO (http://bioinfo.cau.edu.cn/agriGO/) [90] using “suggested backgrounds” as references. These backgrounds were the GO annotation of whole gene sets of organisms. For Brachypodium, sorghum and foxtail millet, only one background was available. For rice and maize, we chose the annotation labeled as “MSU 7.0 nonTE” and “*Zea mays* ssp.”, respectively. In all analyses, statistical tests were performed using the Fisher exact test and the multi-test adjustment method according to Yekutieli [91]; the significance level was set to 0.05; and complete GO was chosen as gene ontology type.

### Expression pattern analysis

Rice expression data were downloaded from PLEXdb (http://www.plexdb.org/). Normalized expression data from various experiments (Table S6) were extracted for early embryogenesis and germ line cells. Details of these experiments can be found at the PLEXdb website under the “Expression Atlases” link. We identified probes corresponding to the rice genes exhibiting intron turnover with the “Gene List Suite” tool (http://www.plexdb.org/modules/glSuite/gl_main.php).

## Supporting Information

Figure S1 Histogram of the number of genes in OrthoMCL clusters.
(TIF)

Figure S2 Examples of enhancing the resolution of intron loss/gain events by adding outgroup data. “*P” (presence) or “*A” (absence) after a gene name indicates the status of the P/A intron(s) in that gene. (a) and (b): History with one event. In (a), loss in branch *bep* (black “-”) or gain in its sister branch (white “+”) are equally possible. BEP is an abbreviation representing the clade of grass subfamilies Bambusoideae, Erhartoideae and Pooideae. In (b), adding Macu genes suggests loss at branch *bep* (black “-”) is the unique most parsimonious reconstruction. (c) and (d), (e) and (f): History with recurrent events. In (c), parsimonious reconstruction provides two possible histories with 2 events: (1) the ancestral state of the intron is absent; 1 gain in Osat (black “+”) and 1 gain in Sita (black “+”); (2) the ancestral state of the intron is absent; 1 gain in common ancestor of Zmay, Sbic, Sita and Osat genes (white “+”) followed by 1 loss in Andropogoneae (white “-”). In (d), orthologous genes in Macu, Smoe and Ppat suggest the ancestral state of the intron is presence with 3 independent losses, in Atha, Andropogoneae and Bdis (three black “-”). In (e), parsimonious reconstruction provides three possible histories with 2 events: (1) the ancestral state of the intron is present; 1 loss in Atha and 1 loss in Sita (two black “-”); (2) the ancestral state of the intron is absent; 1 gain in grasses (white circle) followed by 1 loss in Sita (black circle); (3) the ancestral state of the intron is absent; 1 gain in the common ancestor of the Andropogoneae and 1 gain in the common ancestor of the BEP clade (two white “+”). In (f), adding Macu genes indicates that the ancestral state of the intron is presence, and history (1) with two recurrent losses is the unique most parsimonious reconstruction (two black “-”). Different from (d), the ancestral status in (e) is indicated by paralogous genes because orthologous genes in non-angiosperms are not detected.
(TIF)

Figure S3 An example of the correction of misconstructed intron loss and/or gain events by adding out-group data. (a): When only genes from the six genomes (five grasses and Arabidopsis) are included in the analysis, parsimony suggests that the ancestral state of the intron is absent and 2 gain events occurred, independently in Osat and at the base of the Andropogoneae (two white “+”). (b): Once the more distant Macu data were included, parsimonious reconstruction supports a model in which the ancestral state was presence and recurrent independent losses in Atha, Bdis, Sita and Macu lineages (four black “-”). Any reconstruction involving intron gain requires a greater number of events.
(TIF)

Figure S4 An example of event counting in paralogs in terminal branches.
(TIF)

Figure S5 Relationship of recurrent intron loss events. (a): 2 loss events in orthologous genes. (b): 2 loss events in paralogous genes. (c): 3 loss events in orthologous and paralogous genes. The 2 losses in Osat and Sita are in orthologous genes, while they and the gene with intron loss in Bdis are paralogs. Branches where events happened are marked by black “-”.
(TIF)

Figure S6 Lineage differentiation of events. The branch length of the species tree represents evolutionary time. Branch length of the species tree were adopted from previous publications (see Methods in the main text). (a): The frequencies (number of events per million years (MY)) and rates (number of events per intron per year) of single loss. (b): The frequencies and rates of all intron turnovers.
(TIF)

Figure S7 The size distribution of exons bounded by two neighboring PA intron groups. Density (Y-axis) refers to the frequency density.
(TIF)

Figure S8 Distribution of intron turnover in affected genes. CDS sequence length is normalized to 1 and intron positions in it are scaled accordingly. Headings represent categories of intron groups. Loss: recurrent and single loss groups; SingleLoss: single loss groups; Single: single loss and single gain groups; SingleGain: single gain groups. Error bars in each histogram represent one sd from interval mean values (circle), where mean and sd are calculated by resampling with replacement (1000 times) from the whole intron set.
(TIF)

Figure S9 Distribution of PA intron groups in chromosomes. Short arm and long arm of chromosome are each normalized to 1. Centromere is located at 0 and the short and long arm termini are at -1 and 1, respectively. Headings represent categories of intron groups. Loss: recurrent and single loss groups; SingleLoss: single loss groups; Single: single loss and single gain groups; SingleGain: single gain groups. Error bars in each histogram represent one sd from interval mean values (circle), where mean and sd are calculated by resampling with replacement (1000 times) from the whole intron set.
(TIF)

Figure S10 Potential intron loss hotspots. The Y-axis values are calculated as the density of genes with recurrently lost introns divided by the density of the entire gene set.
(TIF)

Figure S11 Directed acyclic graphs of significant GO terms. Inside the boxes for significant terms is information including: GO term, adjusted p-value, GO description, item number mapping the GO in the query list and background, and total number of query list and background. When the adjusted p-value of a term is higher than the cutoff (here 0.05), only GO information is given. The significance of terms is indicated with color intensity: Terms that are more significant have darker colors (see manul of AgriGO for details: http://bioinfo.cau.edu.cn/agriGO/manual.php).
(PDF)

Figure S12 Distribution of the size of genes with and without intron loss. Gene size is calculated as the length of the genomic region between the translational start and stop codon. The sizes of genes that underwent intron loss are represented by the size of their orthologous *Vitis* genes. Density (Y-axis) refers to the frequency density.
(TIF)

Figure S13 Frequency of intron loss (Y-axis) versus total number of introns in the gene (X-axis). The values on the Y-axis are derived from a simple calculation of the number of intron losses for this category of gene divided by the number of introns in all genes with that number of introns (e.g., one, two, three, etc.). Error bars represent sd from interval mean values (circle), where mean and sd are calculated by resampling with replacement (1000 times) from all of the genes that occur in resolved PA and conserved intron groups.
(TIF)

Figure S14 Distribution of the size of introns that underwent single or recurrent loss events. The sizes of lost introns are represented by the intron size of their closest intron-containing sister lineage in the gene tree. Density (Y-axis) refers to the frequency density.
(TIF)

Figure S15 Identification of an intron group in the CDS alignment of genes belonging to an OrthoMCL cluster. Gene A is a member gene of this cluster. An intron expands between x+1 and y-1 in gene A. Coordinates x and y correspond to i and i+1 in its CDS (CDS A), so a break point is found at (i,i+1) in CDS A. The mapping between genomic coordinates and CDS coordinates is based on GFF files. In the CDS alignment, corresponding positions of this break point, i.e. (j,j+1), are located. If some member genes have no intron at position(j, j+1) in the alignment, an intron polymorphism is observed. We called this polymorphic intron site a presence/absence (PA) intron group candidate. If flanking exon sequences of (j, j+1) are well-aligned (yellow block; quality of the alignment is estimated by comparison of each exon with the consensus sequence of the alignment), an intron group is identified. In the top right diagram, blue blocks represent the last and first base in flanking exon k and k+1. Solid lines indicate x corresponds to i and y to i+1. Light aqua blocks indicate the same region in the gene, CDS and alignment.
(TIF)

Figure S16 Raw read verification of intron groups. Genes in the PA intron group are compared to raw reads. At the target intron group position, if the intron is present, both the 5′ and 3′ exon-intron junctions must be covered by reads (black bars); if the intron is absent, the target position is also required to be covered by reads (yellow bars). Flanking regions of the target intron group are shown as white lines and their conservation is indicated by blue blocks.
(TIF)

Table S1 Summary of detected intron losses and gains in the 179 single-gene OrthoMCL clusters.
(DOCX)

Table S2 Number of loss events in recurrent loss intron groups.
(DOCX)

Table S3 The 10 PA intron groups that appear to have originated by retroposition or whole-gene cDNA conversion.
(DOCX)

Table S4 Number of genes with more than 1 PA intron.
(DOCX)

Table S5 Number of adjacent loss intron pairs in a gene.
(DOCX)

Table S6 Developmental stages of early embryogenesis and pollen used in expression analysis.
(DOCX)

Table S7 G+C richness of recurrent loss introns, PA introns, conserved introns and their flanking exons.
(DOCX)

Table S8 Ranking of dinucleotide frequencies for PA introns, conserved introns, flanking exons and the entire genome.
(DOCX)

Table S9 Dinucleotide frequencies of introns and flanking exons.
(DOCX)

Table S10 Differences in TG/CG ratios between conserved, PA and recurrent loss introns.
(DOCX)

Table S11 Intron-exon boundaries of introns with and without EST support.
(DOCX)

Table S12 Details for all 745 gene clusters with intron absence polymorphism.
(XLSX)
