# Supplementary figures and images for: Recurrent Loss of Specific Introns during Angiosperm Evolution
Source: PLoS Genet. 2014 Dec 4;10(12):e1004843. doi: 10.1371/journal.pgen.1004843 (PMC4256211; doi:10.1371/journal.pgen.1004843)

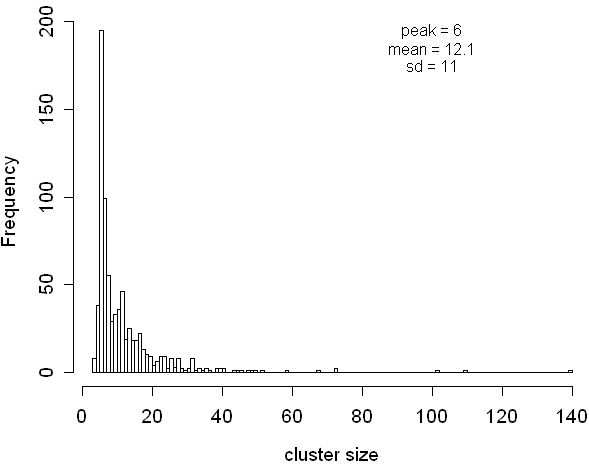

Supplement: Figure S1 — Histogram of the number of genes in OrthoMCL clusters. (TIF) [file pgen.1004843.s001.tif]

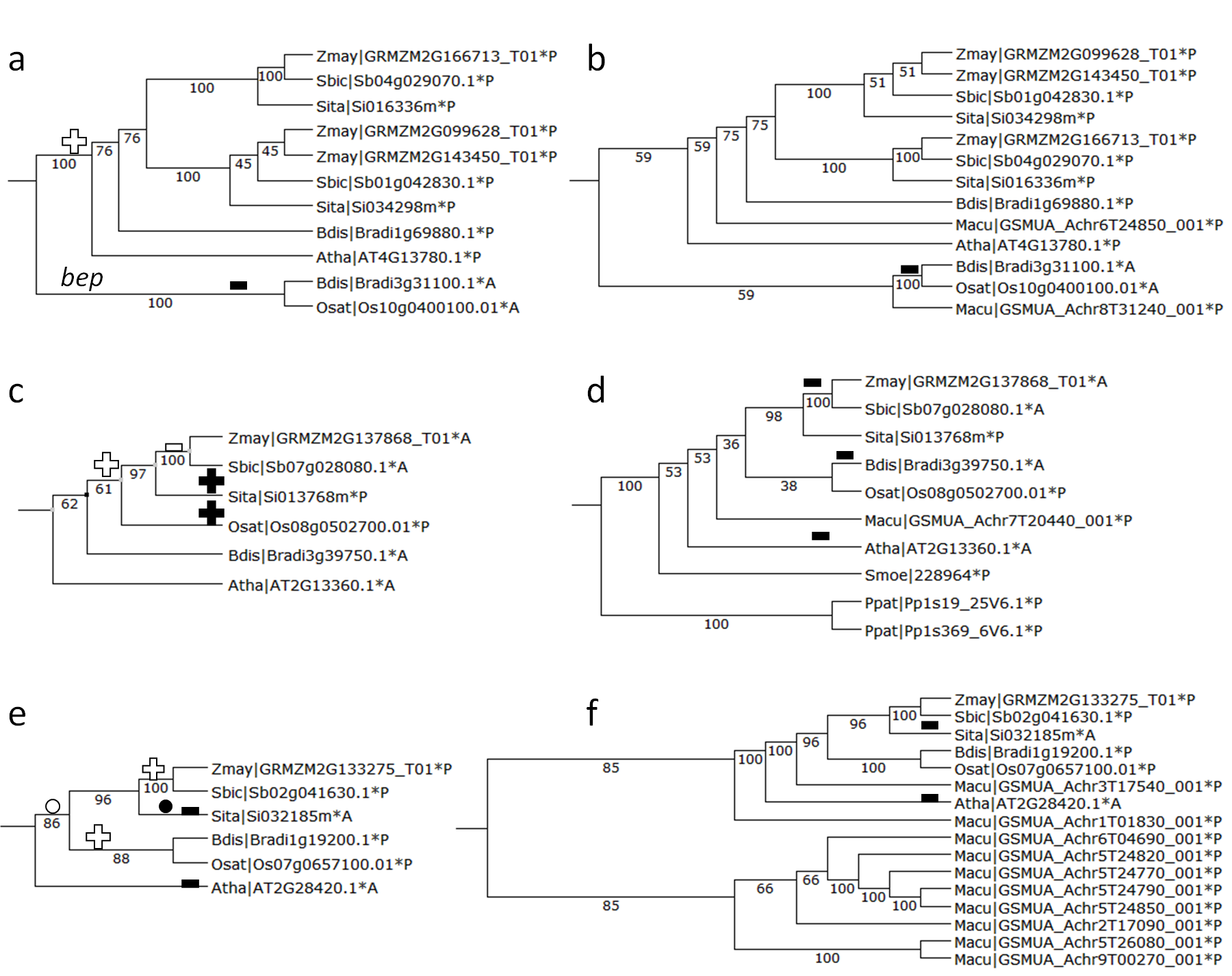

Supplement: Figure S2 — Examples of enhancing the resolution of intron loss/gain events by adding outgroup data. “*P” (presence) or “*A” (absence) after a gene name indicates the status of the P/A intron(s) in that gene. (a) and (b): History with one event. In (a), loss in branch bep (black “-”) or gain in its sister branch (white “+”) are equally possible. BEP is an abbreviation representing the clade of grass subfamilies Bambusoideae, Erhartoideae and Pooideae. In (b), adding Macu genes suggests loss at branch bep (black “-”) is the unique most parsimonious reconstruction. (c) and (d), (e) and (f): History with recurrent events. In (c), parsimonious reconstruction provides two possible histories with 2 events: (1) the ancestral state of the intron is absent; 1 gain in Osat (black “+”) and 1 gain in Sita (black “+”); (2) the ancestral state of the intron is absent; 1 gain in common ancestor of Zmay, Sbic, Sita and Osat genes (white “+”) followed by 1 loss in Andropogoneae (white “-”). In (d), orthologous genes in Macu, Smoe and Ppat suggest the ancestral state of the intron is presence with 3 independent losses, in Atha, Andropogoneae and Bdis (three black “-”). In (e), parsimonious reconstruction provides three possible histories with 2 events: (1) the ancestral state of the intron is present; 1 loss in Atha and 1 loss in Sita (two black “-”); (2) the ancestral state of the intron is absent; 1 gain in grasses (white circle) followed by 1 loss in Sita (black circle); (3) the ancestral state of the intron is absent; 1 gain in the common ancestor of the Andropogoneae and 1 gain in the common ancestor of the BEP clade (two white “+”). In (f), adding Macu genes indicates that the ancestral state of the intron is presence, and history (1) with two recurrent losses is the unique most parsimonious reconstruction (two black “-”). Different from (d), the ancestral status in (e) is indicated by paralogous genes because orthologous genes in non-angiosperms are not detected. (TIF) [file pgen.1004843.s002.tif]

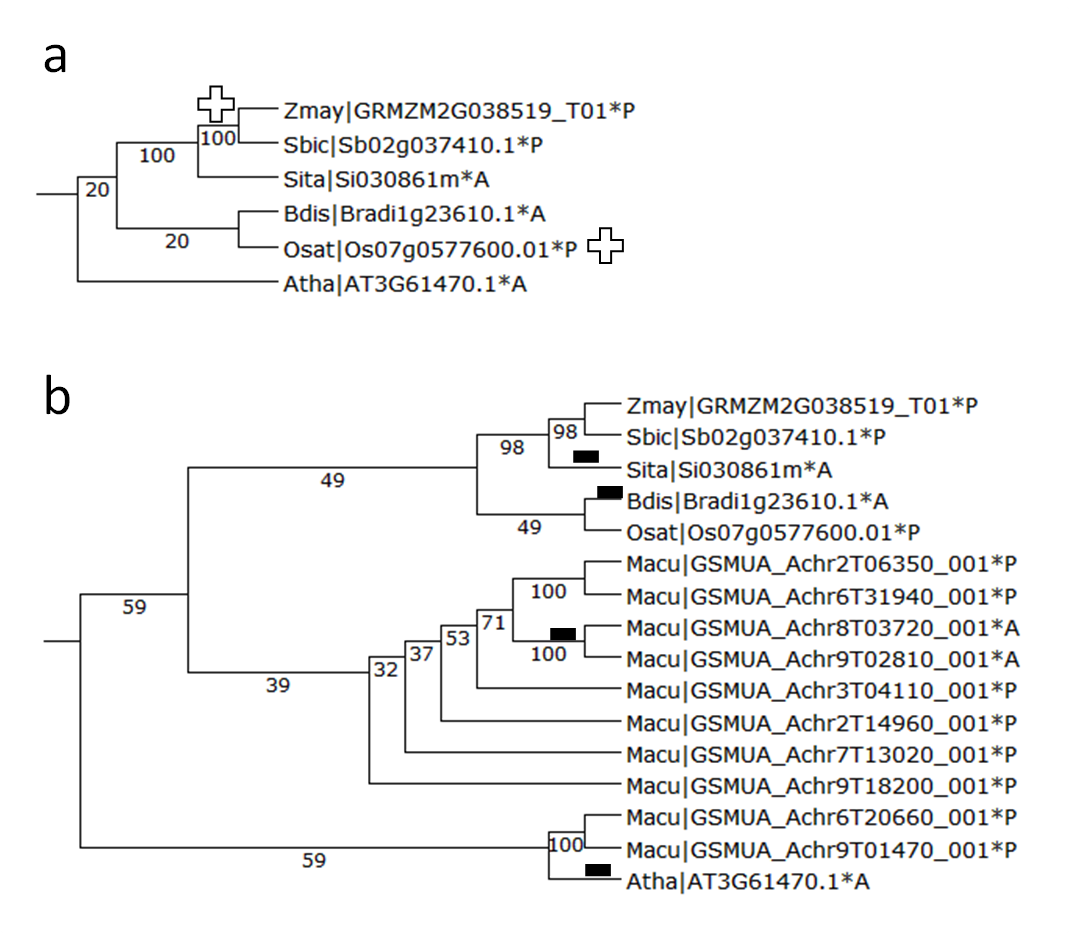

Supplement: Figure S3 — An example of the correction of misconstructed intron loss and/or gain events by adding out-group data. (a): When only genes from the six genomes (five grasses and Arabidopsis) are included in the analysis, parsimony suggests that the ancestral state of the intron is absent and 2 gain events occurred, independently in Osat and at the base of the Andropogoneae (two white “+”). (b): Once the more distant Macu data were included, parsimonious reconstruction supports a model in which the ancestral state was presence and recurrent independent losses in Atha, Bdis, Sita and Macu lineages (four black “-”). Any reconstruction involving intron gain requires a greater number of events. (TIF) [file pgen.1004843.s003.tif]

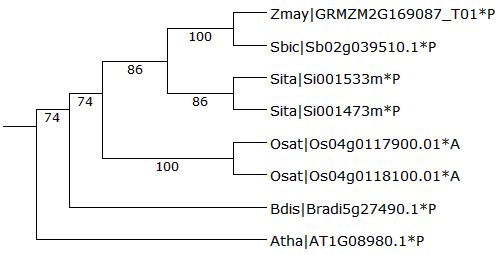

Supplement: Figure S4 — An example of event counting in paralogs in terminal branches. (TIF) [file pgen.1004843.s004.tif]

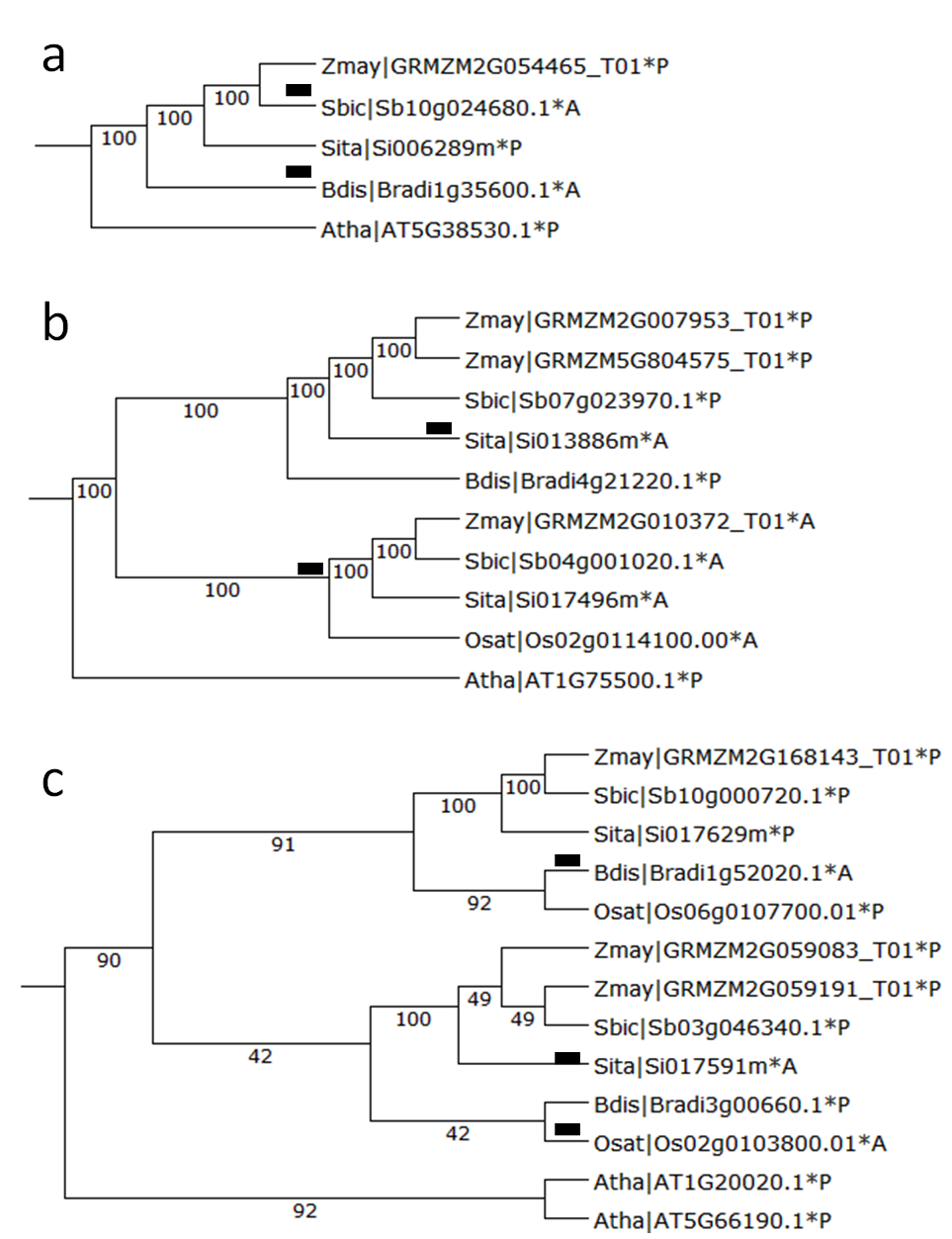

Supplement: Figure S5 — Relationship of recurrent intron loss events. (a): 2 loss events in orthologous genes. (b): 2 loss events in paralogous genes. (c): 3 loss events in orthologous and paralogous genes. The 2 losses in Osat and Sita are in orthologous genes, while they and the gene with intron loss in Bdis are paralogs. Branches where events happened are marked by black “-”. (TIF) [file pgen.1004843.s005.tif]

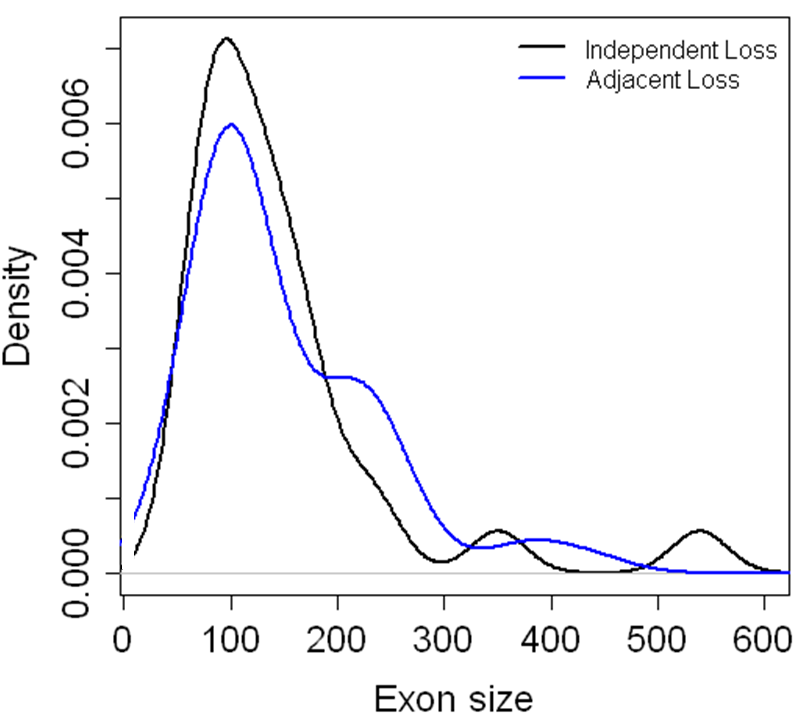

Supplement: Figure S7 — The size distribution of exons bounded by two neighboring PA intron groups. Density (Y-axis) refers to the frequency density. (TIF) [file pgen.1004843.s007.tif]

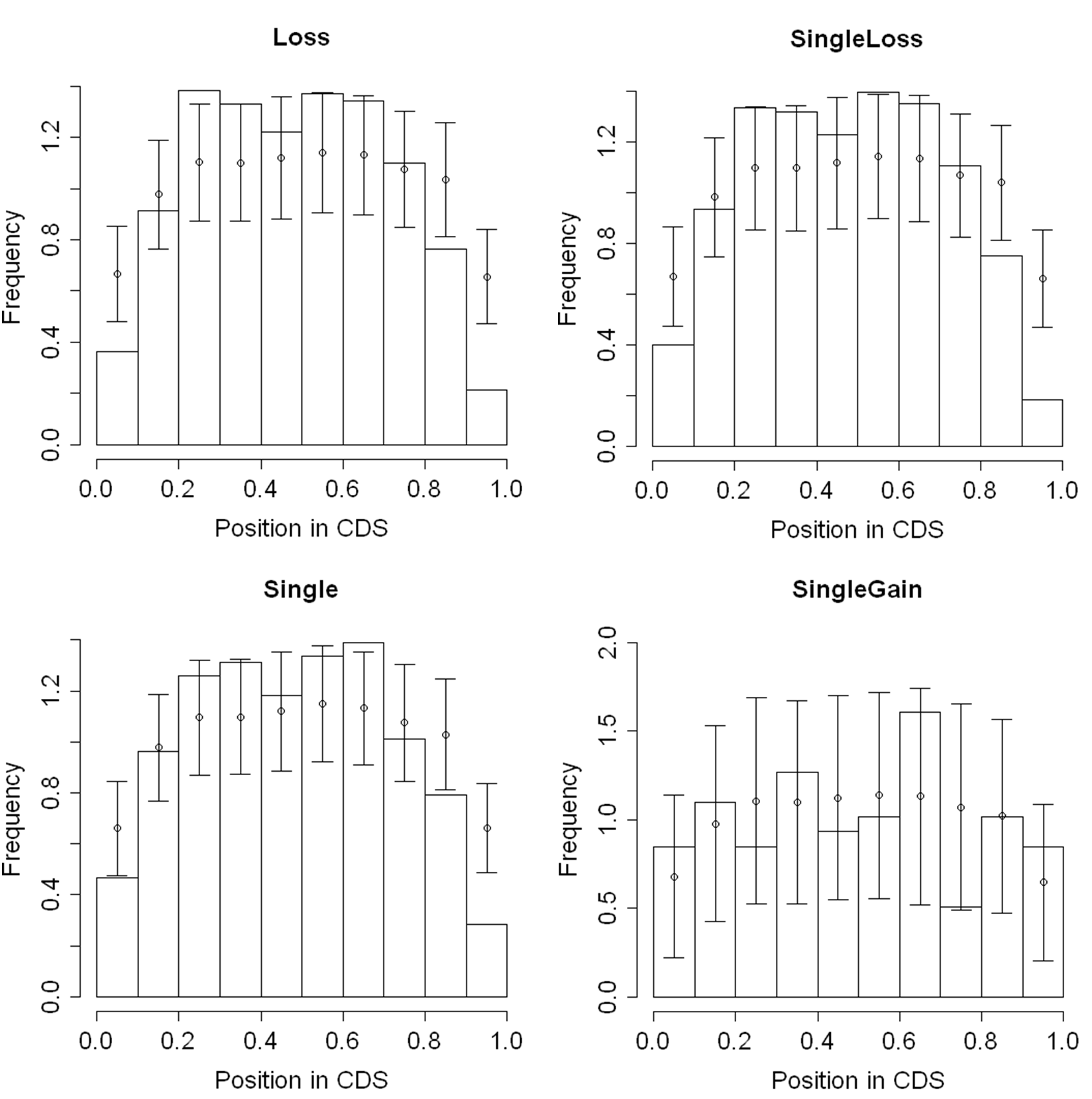

Supplement: Figure S8 — Distribution of intron turnover in affected genes. CDS sequence length is normalized to 1 and intron positions in it are scaled accordingly. Headings represent categories of intron groups. Loss: recurrent and single loss groups; SingleLoss: single loss groups; Single: single loss and single gain groups; SingleGain: single gain groups. Error bars in each histogram represent one sd from interval mean values (circle), where mean and sd are calculated by resampling with replacement (1000 times) from the whole intron set. (TIF) [file pgen.1004843.s008.tif]

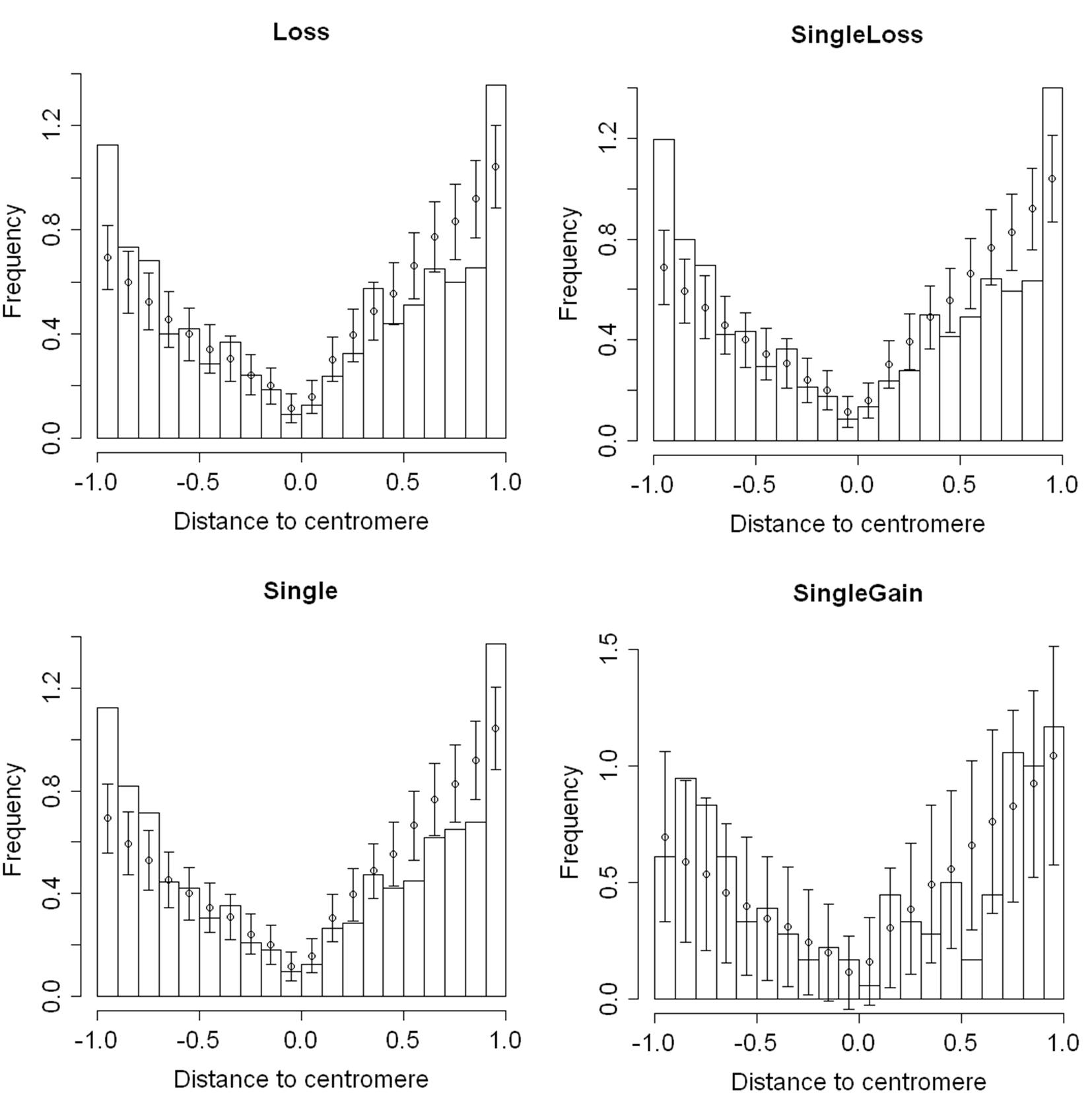

Supplement: Figure S9 — Distribution of PA intron groups in chromosomes. Short arm and long arm of chromosome are each normalized to 1. Centromere is located at 0 and the short and long arm termini are at -1 and 1, respectively. Headings represent categories of intron groups. Loss: recurrent and single loss groups; SingleLoss: single loss groups; Single: single loss and single gain groups; SingleGain: single gain groups. Error bars in each histogram represent one sd from interval mean values (circle), where mean and sd are calculated by resampling with replacement (1000 times) from the whole intron set. (TIF) [file pgen.1004843.s009.tif]

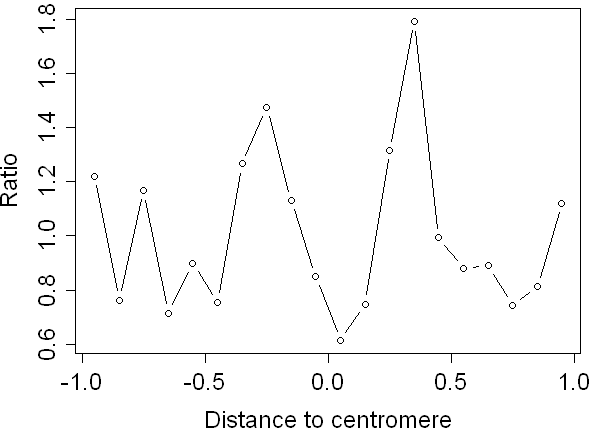

Supplement: Figure S10 — Potential intron loss hotspots. The Y-axis values are calculated as the density of genes with recurrently lost introns divided by the density of the entire gene set. (TIF) [file pgen.1004843.s010.tif]

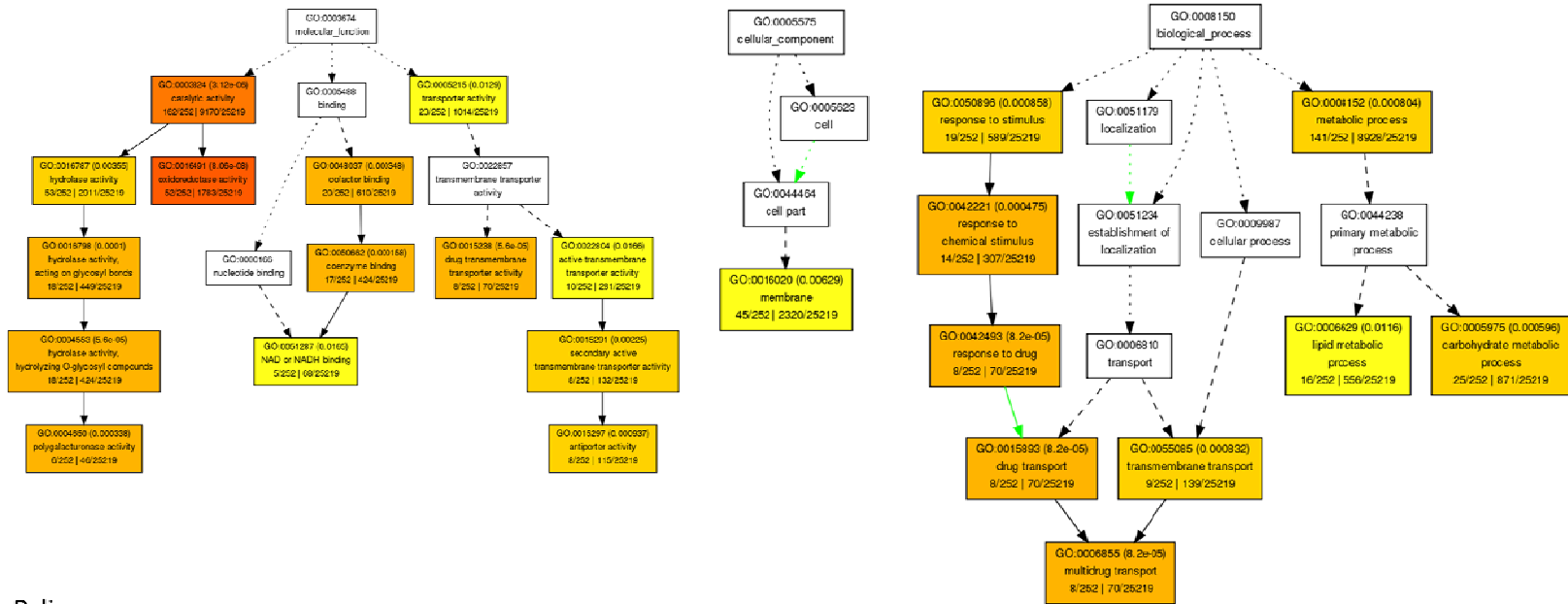

Bdis

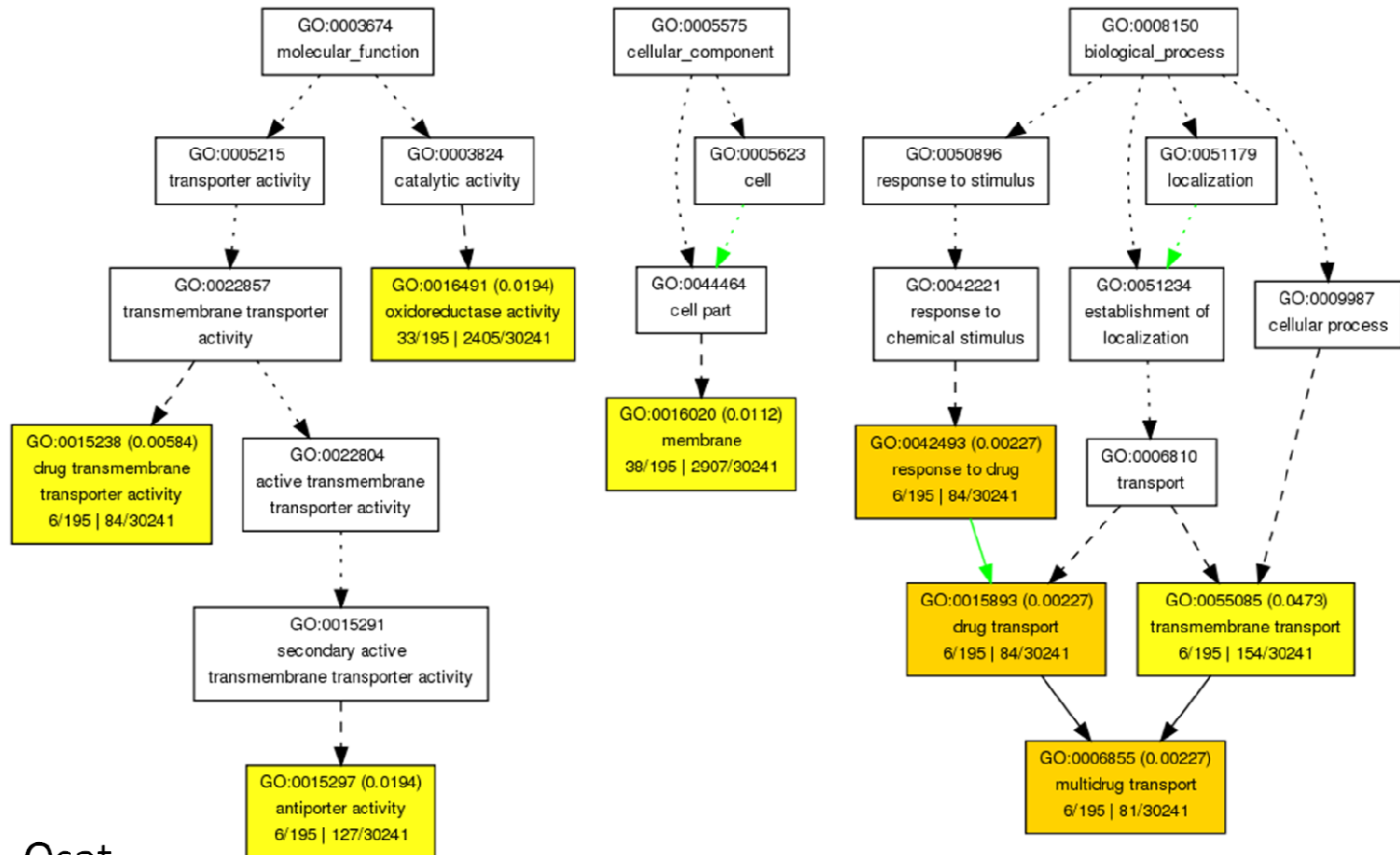

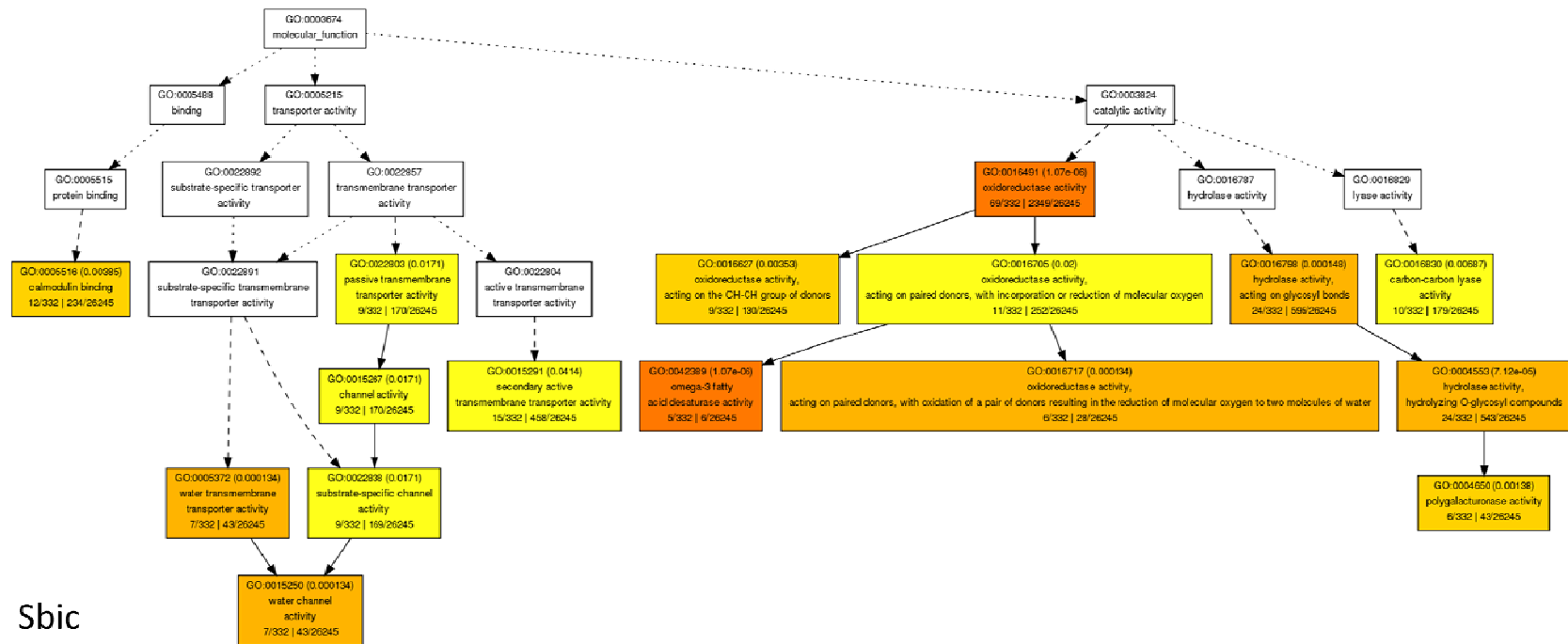

Sbic

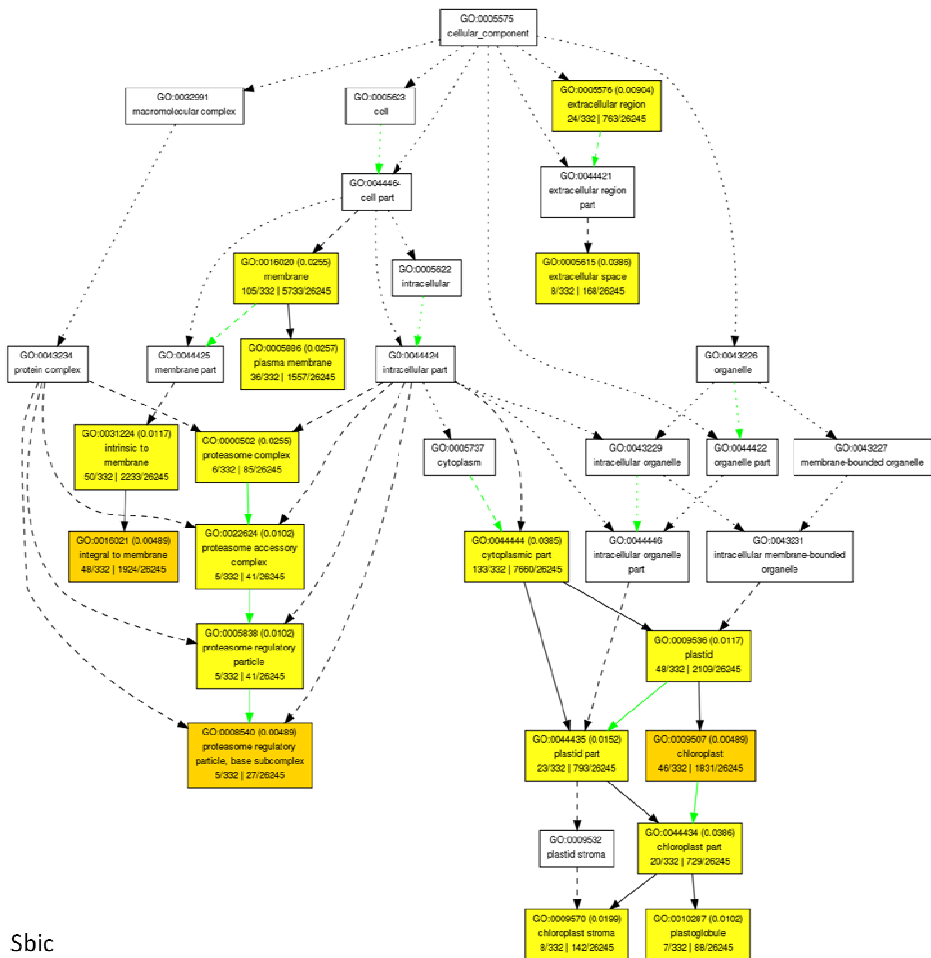

Sbic

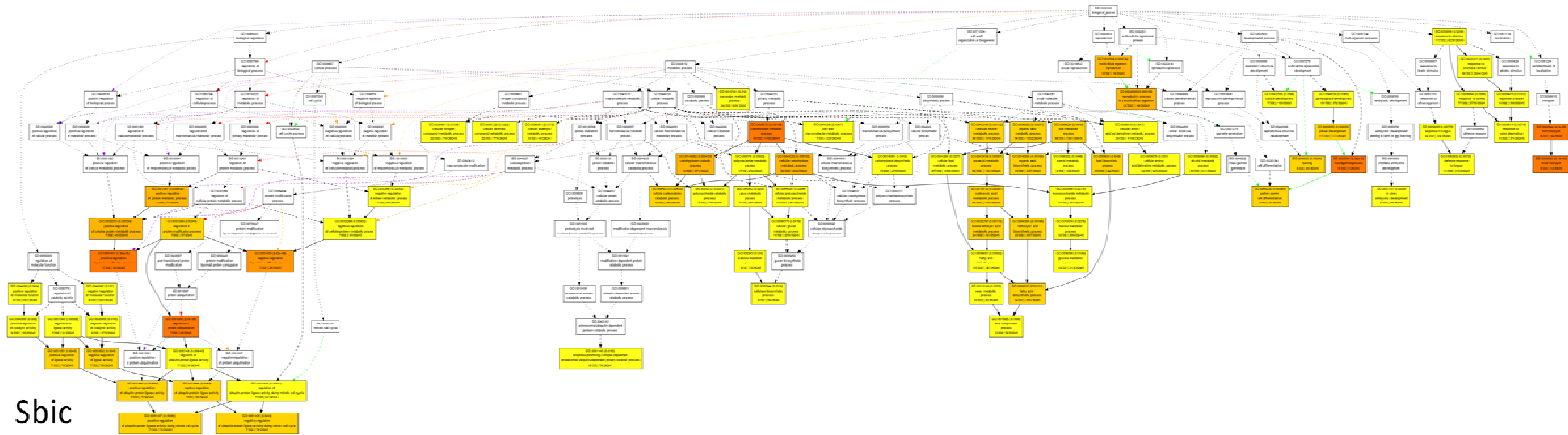



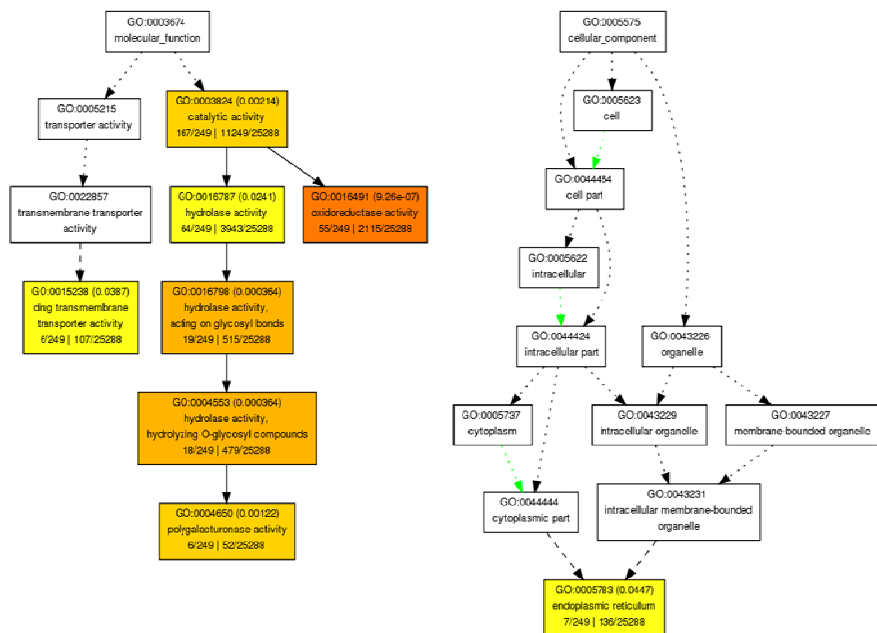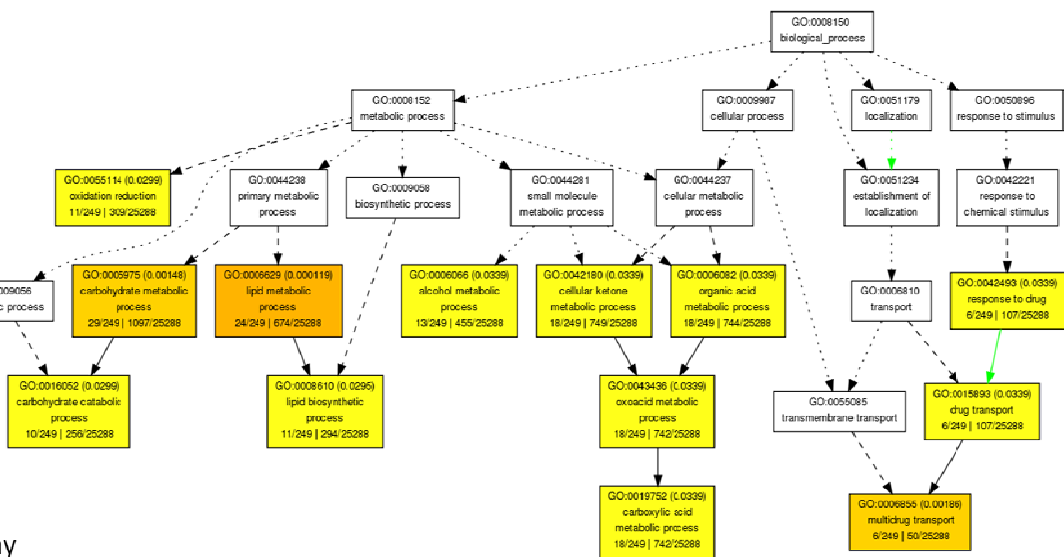

Supplement: Figure S11 — Directed acyclic graphs of significant GO terms. Inside the boxes for significant terms is information including: GO term, adjusted p-value, GO description, item number mapping the GO in the query list and background, and total number of query list and background. When the adjusted p-value of a term is higher than the cutoff (here 0.05), only GO information is given. The significance of terms is indicated with color intensity: Terms that are more significant have darker colors (see manul of AgriGO for details: http://bioinfo.cau.edu.cn/agriGO/manual.php). (PDF) [file pgen.1004843.s011.pdf]

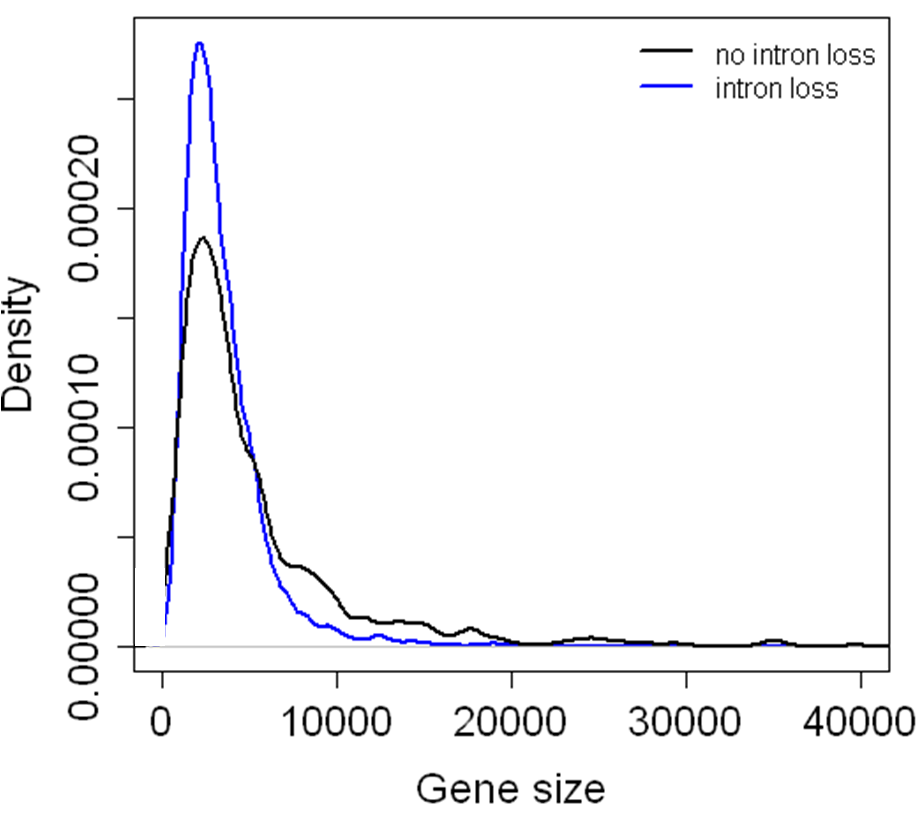

Supplement: Figure S12 — Distribution of the size of genes with and without intron loss. Gene size is calculated as the length of the genomic region between the translational start and stop codon. The sizes of genes that underwent intron loss are represented by the size of their orthologous Vitis genes. Density (Y-axis) refers to the frequency density. (TIF) [file pgen.1004843.s012.tif]

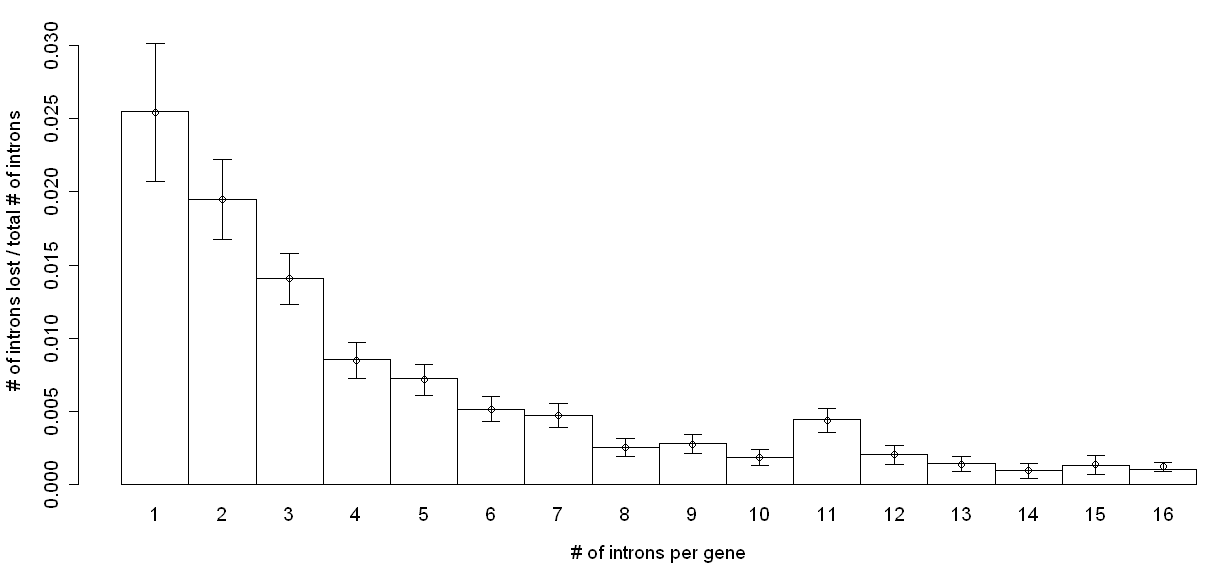

Supplement: Figure S13 — Frequency of intron loss (Y-axis) versus total number of introns in the gene (X-axis). The values on the Y-axis are derived from a simple calculation of the number of intron losses for this category of gene divided by the number of introns in all genes with that number of introns (e.g., one, two, three, etc.). Error bars represent sd from interval mean values (circle), where mean and sd are calculated by resampling with replacement (1000 times) from all of the genes that occur in resolved PA and conserved intron groups. (TIF) [file pgen.1004843.s013.tif]

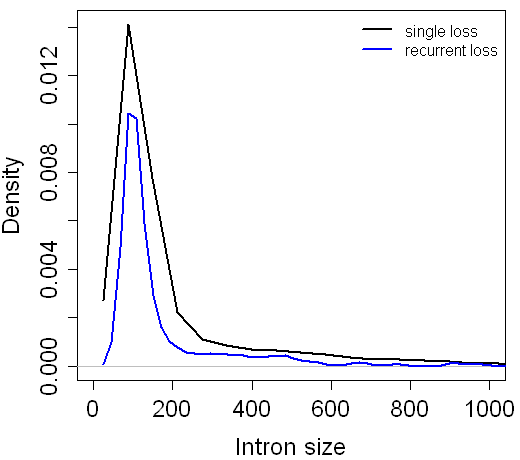

Supplement: Figure S14 — Distribution of the size of introns that underwent single or recurrent loss events. The sizes of lost introns are represented by the intron size of their closest intron-containing sister lineage in the gene tree. Density (Y-axis) refers to the frequency density. (TIF) [file pgen.1004843.s014.tif]

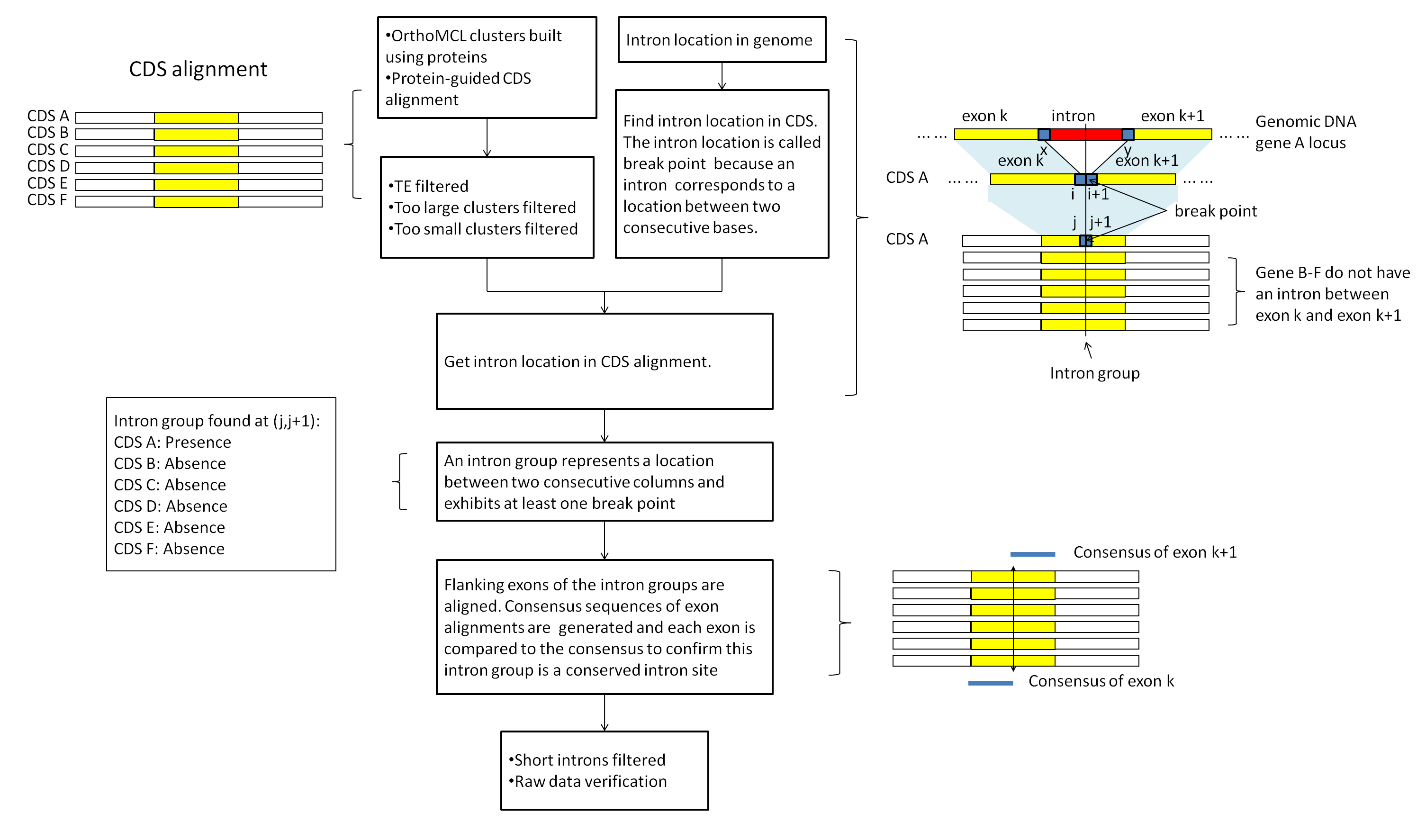

Supplement: Figure S15 — Identification of an intron group in the CDS alignment of genes belonging to an OrthoMCL cluster. Gene A is a member gene of this cluster. An intron expands between x+1 and y-1 in gene A. Coordinates x and y correspond to i and i+1 in its CDS (CDS A), so a break point is found at (i,i+1) in CDS A. The mapping between genomic coordinates and CDS coordinates is based on GFF files. In the CDS alignment, corresponding positions of this break point, i.e. (j,j+1), are located. If some member genes have no intron at position(j, j+1) in the alignment, an intron polymorphism is observed. We called this polymorphic intron site a presence/absence (PA) intron group candidate. If flanking exon sequences of (j, j+1) are well-aligned (yellow block; quality of the alignment is estimated by comparison of each exon with the consensus sequence of the alignment), an intron group is identified. In the top right diagram, blue blocks represent the last and first base in flanking exon k and k+1. Solid lines indicate x corresponds to i and y to i+1. Light aqua blocks indicate the same region in the gene, CDS and alignment. (TIF) [file pgen.1004843.s015.tif]
