# Supplementary material for: Recurrent Loss of Specific Introns during Angiosperm Evolution
Source: PLoS Genet. 2014 Dec 4;10(12):e1004843. doi: 10.1371/journal.pgen.1004843 (PMC4256211; doi:10.1371/journal.pgen.1004843)
Supplement: Table S1 — Summary of detected intron losses and gains in the 179 single-gene OrthoMCL clusters. (DOCX) [file pgen.1004843.s017.docx]

Table S1: Summary of detected intron losses and gains in the 179 single-gene OrthoMCL clusters.

| Category |  | Number of intron groups | Number of events | Number of affected genes^(1)^ |
| --- | --- | --- | --- | --- |
| Single | Gain | 25 | 25 | 31 |
|  | Loss | 145 | 145 | 210 |
| Recurrent | Gain | 0 | 0 | 0 |
|  | Loss | 18 | 37 | 39 |
|  | Mix^(2)^ | 4 | 8 | 20 |
|  | Unresolved | 20 | NA | NA |
| Total |  | 212 | 215 | 300 |

(1) One gene may be counted several times since it may exhibit several categories of events

(2) Loss followed by gain or gain followed by loss according to the most parsimonious reconstruction
