# Supplementary material for: Recurrent Loss of Specific Introns during Angiosperm Evolution
Source: PLoS Genet. 2014 Dec 4;10(12):e1004843. doi: 10.1371/journal.pgen.1004843 (PMC4256211; doi:10.1371/journal.pgen.1004843)
Supplement: Table S2 — Number of loss events in recurrent loss intron groups. (DOCX) [file pgen.1004843.s018.docx]

Table S2: Number of loss event in recurrent loss intron groups.

| Number of recurrent loss events per group | Number of recurrent loss intron groups with this many recurrent losses of the same introns |
| --- | --- |
| 2 | 82 |
| 3 | 7 |
| 4 | 2 |
| 6 | 1 |
| 7 | 1 |
