# Supplementary material for: Recurrent Loss of Specific Introns during Angiosperm Evolution
Source: PLoS Genet. 2014 Dec 4;10(12):e1004843. doi: 10.1371/journal.pgen.1004843 (PMC4256211; doi:10.1371/journal.pgen.1004843)
Supplement: Table S3 — The 10 PA intron groups that appear to have originated by retroposition or whole-gene cDNA conversion. (DOCX) [file pgen.1004843.s019.docx]

Table S3: The 10 PA intron groups that appear to have originated by retroposition or whole-gene cDNA conversion.

| Group ID | In synteny block | Number of introns* | Functional Annotation | Event lineage | Mechanism | Member genes with single exon |
| --- | --- | --- | --- | --- | --- | --- |
| G5A_10034 | Andropogoneae | 20 | Starch branching enzyme | Panicoid | RTMIL** | Sbic\|Sb03g027310.1,Zmay\|GRMZM2G005298_T01 |
| G5A_1242 | grass | 11 | Polynucleotide adenylyltransferase activity (Blast2GO) | grass | RTMIL | Bdis\|Bradi5g19750.1,Sbic\|Sb06g026810.1,Sita\|Si012029m,Zmay\|GRMZM2G032554_T01 |
| G5A_11874 | No | 17 | Acetylglucosaminyltransferase activity | Bdis | Retroposition | Bdis\|Bradi1g08940.1 |
| G5A_1315 | No | 8 | Ructokinase activity, mannokinase activity, glucokinase activity, ATPase activity (Blast2GO) | Osat | Retroposition | Osat\|Os07g0446800.00 |
| G5A_1828 | No | 8 | Similar to signal recognition particle 54 kDa protein | Panicoid | Retroposition | Sbic\|Sb01g014030.1,Sita\|Si038716m |
| G5A_2491 | No | 9 | Beclin-1, putative, expressed | Osat | Retroposition | Osat\|Os03g0258500.00 |
| G5A_4386 | No | 8 | GTPase activity (Blast2GO) | grass | Retroposition | Bdis\|Bradi3g10590.1,Sbic\|Sb09g003220.1 |
| G5A_5589 | No | 13 | RRM domain containing protein similar to putative peptidyl-prolyl cis-trans isomerase, | Bdis | Retroposition | Bdis\|Bradi2g41160.1 |
| G5A_5224 | No | 21 | Syntaxin-3 binding, syntaxin-2 binding (Blast2GO) | Bdis | Retroposition | Bdis\|Bradi1g75720.1 |
| G5A_1017 | No | 13 | Various Functions | grass | Retroposition | See Fig. S6 for details |

* Intron number of intron-containing member genes

** RTMIL: Reverse Transcriptase-Mediated Intron Loss
