# Supplementary material for: Recurrent Loss of Specific Introns during Angiosperm Evolution
Source: PLoS Genet. 2014 Dec 4;10(12):e1004843. doi: 10.1371/journal.pgen.1004843 (PMC4256211; doi:10.1371/journal.pgen.1004843)
Supplement: Table S4 — Number of genes with more than 1 PA intron. (DOCX) [file pgen.1004843.s020.docx]

Table S4: Number of genes with more than 1 PA intron.

| Number of PA intron groups | Number of OrthoMCL clusters with this many different PA introns |
| --- | --- |
| 2 | 73 |
| 3 | 40 |
| 4 | 7 |
| 5 | 5 |
| 6 | 4 |
| 7 | 1 |
| SUM | 130 |
