# Supplementary material for: Recurrent Loss of Specific Introns during Angiosperm Evolution
Source: PLoS Genet. 2014 Dec 4;10(12):e1004843. doi: 10.1371/journal.pgen.1004843 (PMC4256211; doi:10.1371/journal.pgen.1004843)
Supplement: Table S5 — Number of adjacent loss intron pairs in a gene. (DOCX) [file pgen.1004843.s021.docx]

Table S5: Number of adjacent loss intron pairs in a gene.

| Number of adjacent loss intron groups | Number of OrthoMCL clusters with this many adjacent intron losses |
| --- | --- |
| 2 | 29 |
| 3 | 5 |
| 4 | 4 |
| 5 | 2 |
