# Supplementary material for: Recurrent Loss of Specific Introns during Angiosperm Evolution
Source: PLoS Genet. 2014 Dec 4;10(12):e1004843. doi: 10.1371/journal.pgen.1004843 (PMC4256211; doi:10.1371/journal.pgen.1004843)
Supplement: Table S6 — Developmental stages of early embryogenesis and pollen used in expression analysis. (DOCX) [file pgen.1004843.s022.docx]

Table S6: Developmental stages of early embryogenesis and pollen used in expression analyses (http://www.plexdb.org/).

| Study | Tissue | Experiment ID | Developmental stage |
| --- | --- | --- | --- |
| Early embryogenesis | Early embryogenesis | OS46 | 1_DAP_embryosac_bottom |
|  |  | OS46 | 1_DAP_embryosac_top |
|  |  | OS46 | 2_DAP_embryosac_bottom |
|  |  | OS46 | 2_DAP_embryosac_top |
|  |  | OS46 | 3_DAP_embryosac_bottom |
|  |  | OS46 | 3_DAP_embryosac_top |
|  |  | OS46 | 4_DAP_embryosac_bottom |
|  |  | OS46 | 4_DAP_embryosac_top |
|  |  | OS8 | emb6D_rep1 |
|  |  | OS89 | Embryo_at_9DAF |
|  |  | OS89 | Embryo_at_12DAF |
| Control | Root | OS46 | NA |
|  | Shoot | OS46 | NA |
|  | Young_leaf | OS46 | NA |
| Germ line cell | Pollen | OS63 | bicellular_pollen_1 |
|  |  | OS63 | germinated_pollen_grains_1 |
|  |  | OS63 | mature_pollen_grains_1 |
|  |  | OS63 | tricellular_pollen_1 |
|  |  | OS63 | uninucleate_microspores_1 |
|  |  | OS94 | Sperm_at_anthesis |
| Control | Leaf | OS63 | NA |
|  | Root | OS63 | NA |
|  | callus_cell | OS63 | NA |
