# Supplementary material for: Recurrent Loss of Specific Introns during Angiosperm Evolution
Source: PLoS Genet. 2014 Dec 4;10(12):e1004843. doi: 10.1371/journal.pgen.1004843 (PMC4256211; doi:10.1371/journal.pgen.1004843)
Supplement: Table S7 — G+C richness of recurrent loss introns, PA introns, conserved introns and their flanking exons. (DOCX) [file pgen.1004843.s023.docx]

Table S7: G+C richness of recurrent loss introns, PA introns, conserved introns and their flanking exons.

|  | Recurrent loss | PA | Conserved | All exons or introns |
| --- | --- | --- | --- | --- |
| Upstream exon^(1)^ | 0.55 | 0.54 | 0.47 | 0.55^(2)^ |
| Intron | 0.39 | 0.39 | 0.39 | 0.39^(3)^ |
| Downstream exon^(1)^ | 0.57 | 0.54 | 0.46 | 0.55^(2)^ |
| Genome wide^(4)^ | 0.44 | | | |

(1) 20 bp of exon sequence adjacent to the intron

(2) G**enome-wide** G+C richness **for all exons.**

(3) G**enome-wide** G+C richness **for all introns.**

**(4)** Combined G+C richness **of 5 grass genomes.**
