# Supplementary material for: Recurrent Loss of Specific Introns during Angiosperm Evolution
Source: PLoS Genet. 2014 Dec 4;10(12):e1004843. doi: 10.1371/journal.pgen.1004843 (PMC4256211; doi:10.1371/journal.pgen.1004843)
Supplement: Table S8 — Ranking of dinucleotide frequencies for PA introns, conserved introns, flanking exons and the entire genome. (DOCX) [file pgen.1004843.s024.docx]

Table S8: Ranking of dinucleotide frequencies for PA introns, conserved introns, flanking exons and the entire genome.

| Rank* |  | 1 | 2 | 3 | 4 | 5 | 6 | 7 | 8 | 9 | 10 | 11 | 12 | 13 | 14 | 15 | 16 |
| --- | --- | --- | --- | --- | --- | --- | --- | --- | --- | --- | --- | --- | --- | --- | --- | --- | --- |
| Intron | Recurrent loss | CG | GG | CC | GC | AG | GA | AC | GT | TC | CT | CA | TG | TA | AA | AT | TT |
|  | PA | CG | GG | CC | GC | AC | GA | AG | GT | CA | TC | CT | TA | TG | AA | AT | TT |
|  | Conserved | CG | GG | CC | GC | AC | GA | AG | TC | CA | GT | CT | TA | AA | TG | AT | TT |
| Flanking exon | Exon downstream of recurrent loss intron | TA | TT | AT | AA | GT | AG | AC | CT | TG | TC | GA | CA | CC | CG | GG | GC |
|  | Exon upstream of recurrent loss intron | TA | AA | AT | TT | GT | AG | AC | GA | TG | CA | CT | TC | GG | CC | CG | GC |
|  | Exon downstream of PA intron | TA | TT | AT | AA | GT | AC | CT | AG | TC | CC | CA | TG | GA | CG | GG | GC |
|  | Exon upstream of PA intron | TA | TT | AT | AA | GT | AC | AG | CT | TG | CA | GA | TC | CC | GG | CG | GC |
|  | Exon downstream of conserved intron | CG | TA | CC | AC | GT | TC | GC | GG | CT | AT | TT | CA | AG | AA | GA | TG |
|  | Exon upstream of conserved intron | TA | CG | AC | GT | CC | AT | TC | TT | CT | GG | CA | GC | AG | AA | GA | TG |
| Genome wide |  | CG | GT | AC | GC | GG | CC | TA | AG | CT | GA | TC | TG | CA | AT | TT | AA |

*Frequency of dinucleotides increases with the rank change from 1 to 16
