# Supplementary material for: Recurrent Loss of Specific Introns during Angiosperm Evolution
Source: PLoS Genet. 2014 Dec 4;10(12):e1004843. doi: 10.1371/journal.pgen.1004843 (PMC4256211; doi:10.1371/journal.pgen.1004843)
Supplement: Table S9 — Dinucleotide frequencies of introns and flanking exons. (DOCX) [file pgen.1004843.s025.docx]

Table S9: Dinucleotide frequencies of introns and flanking exons.

|  | Genome wide | Conserved intron | Exon downstream of conserved intron | Exon upstream of conserved intron | PA intron | Exon downstream of PA intron | Exon upstream of PA intron | Recurrent loss intron | Exon downstream of recurrent loss intron | Exon upstream of recurrent loss intron |
| --- | --- | --- | --- | --- | --- | --- | --- | --- | --- | --- |
| A+T richness | 0.53 | 0.61 | 0.53 | 0.50 | 0.61 | 0.44 | 0.42 | 0.61 | 0.42 | 0.41 |
| AA | 0.08 | 0.08 | 0.08 | 0.07 | 0.08 | 0.05 | 0.05 | 0.09 | 0.05 | 0.04 |
| AC | 0.05 | 0.05 | 0.05 | 0.05 | 0.05 | 0.06 | 0.06 | 0.05 | 0.06 | 0.06 |
| AG | 0.06 | 0.05 | 0.07 | 0.07 | 0.05 | 0.06 | 0.06 | 0.05 | 0.06 | 0.05 |
| AT | 0.07 | 0.09 | 0.07 | 0.06 | 0.09 | 0.05 | 0.05 | 0.09 | 0.05 | 0.05 |
| CA | 0.07 | 0.06 | 0.07 | 0.07 | 0.06 | 0.07 | 0.07 | 0.07 | 0.07 | 0.07 |
| CC | 0.06 | 0.04 | 0.05 | 0.06 | 0.04 | 0.07 | 0.08 | 0.04 | 0.07 | 0.08 |
| CG | 0.04 | 0.02 | 0.03 | 0.05 | 0.03 | 0.07 | 0.08 | 0.03 | 0.08 | 0.09 |
| CT | 0.06 | 0.07 | 0.06 | 0.07 | 0.07 | 0.06 | 0.07 | 0.06 | 0.06 | 0.07 |
| GA | 0.06 | 0.05 | 0.08 | 0.07 | 0.05 | 0.07 | 0.07 | 0.05 | 0.07 | 0.06 |
| GC | 0.05 | 0.04 | 0.06 | 0.07 | 0.04 | 0.08 | 0.09 | 0.05 | 0.09 | 0.09 |
| GG | 0.06 | 0.04 | 0.06 | 0.07 | 0.04 | 0.08 | 0.08 | 0.03 | 0.09 | 0.08 |
| GT | 0.05 | 0.06 | 0.05 | 0.05 | 0.06 | 0.05 | 0.05 | 0.06 | 0.05 | 0.05 |
| TA | 0.06 | 0.08 | 0.04 | 0.04 | 0.07 | 0.03 | 0.03 | 0.08 | 0.03 | 0.02 |
| TC | 0.06 | 0.06 | 0.06 | 0.06 | 0.06 | 0.07 | 0.07 | 0.06 | 0.07 | 0.08 |
| TG | 0.07 | 0.08 | 0.08 | 0.08 | 0.08 | 0.07 | 0.07 | 0.08 | 0.07 | 0.06 |
| TT | 0.08 | 0.13 | 0.07 | 0.07 | 0.12 | 0.05 | 0.05 | 0.11 | 0.04 | 0.05 |
| TG/CG | 1.62 | 4.22 | 2.43 | 1.62 | 2.92 | 0.96 | 0.83 | 2.45 | 0.82 | 0.73 |
