# Supplementary material for: Recurrent Loss of Specific Introns during Angiosperm Evolution
Source: PLoS Genet. 2014 Dec 4;10(12):e1004843. doi: 10.1371/journal.pgen.1004843 (PMC4256211; doi:10.1371/journal.pgen.1004843)
Supplement: Table S10 — Differences in TG/CG ratios between conserved, PA and recurrent loss introns. (DOCX) [file pgen.1004843.s026.docx]

Table S10: Difference* in TG/CG ratios between conserved, PA and recurrent loss introns

| Intron category | TG count | CG count | TG/CG |
| --- | --- | --- | --- |
| Conserved | 6,032,257 | 1,431,053 | 4.22 |
| PA | 180,213 | 61,806 | 2.92 |
| Recurrent loss | 21,179 | 8652 | 2.45 |

* The inequality “TG/CG in conserved introns > TG/CG in PA introns > TG/CG in recurrent loss introns” holds at p-value<1e-16, according to Pearson’s Chi-square test with Bonferroni correction.
