# Supplementary material for: Recurrent Loss of Specific Introns during Angiosperm Evolution
Source: PLoS Genet. 2014 Dec 4;10(12):e1004843. doi: 10.1371/journal.pgen.1004843 (PMC4256211; doi:10.1371/journal.pgen.1004843)
Supplement: Table S11 — Intron-exon boundaries of introns with and without EST support. (DOCX) [file pgen.1004843.s027.docx]

Table S11: Intron-exon boundaries of introns with and without EST support

| Flanking  dinucleotides | # of introns in Conserved intron groups | | # of introns in PA intron groups | |
| --- | --- | --- | --- | --- |
|  | EST support | No EST support | EST support | No EST support |
| GT..AG | 189,756 | 6841 | 7798 | 48 |
| GC..AG | 1224 | 40 | 79 | 3 |
| AT..AC | 26 | 0 | 0 | 0 |
